# BMI, Diet and Female Reproductive Factors as Risks for Thyroid Cancer: A Systematic Review

**DOI:** 10.1371/journal.pone.0029177

**Published:** 2012-01-19

**Authors:** Emily Peterson, Prithwish De, Robert Nuttall

**Affiliations:** 1 University of Toronto, Dalla Lana School of Public Health, Division of Epidemiology, Toronto, Ontario, Canada; 2 Canadian Cancer Society, Cancer Control Policy, Toronto, Ontario, Canada; Cardiff University, United Kingdom

## Abstract

**Background:**

Thyroid cancer incidence rates have been increasing worldwide but the reason behind this is unclear. Both the increasing use of diagnostic technologies allowing the detection of thyroid cancer and a true increase in thyroid cancer incidence have been proposed. This review assesses the role of body mass index (BMI), diet, and reproductive factors on the thyroid cancer trend.

**Methods:**

Epidemiologic studies of the selected risk factors up to June 2010 were reviewed and critically assessed.

**Results:**

Among the thirty-seven studies reviewed and despite variation in the risk estimates, most papers supported a small but positive association for BMI (risk estimate range: 1.1–2.3 in males and 1.0–7.4 in females.). Among specific dietary components, there was no consistent association of thyroid cancer risk with iodine intake through fortification (risk estimate range: 0.49–1.6) or fish consumption (risk estimate range 0.6–2.2), nor with diets high in cruciferous vegetables (risk estimate range 0.6–1.9). A small number of studies showed a consistent protective effect of diets high in non-cruciferous vegetable (risk estimate range: 0.71–0.92). Among reproductive factors (pregnancy, parity, number of live births, use of prescription hormones, menstrual cycle regularity, and menopausal status), none were consistently associated with higher thyroid cancer risk.

**Conclusions:**

BMI had the strongest link to thyroid cancer risk among those examined. Detailed examinations of population-level risk factors can help identify and support prevention efforts to reduce the burden of thyroid cancer.

## Introduction

Over the past 30 years many countries have experienced a dramatic rise in the incidence of thyroid cancer, with a study across five continents showing the average increase to be 67% in females and 48% in males between 1973 and 2002 [1]. While the increase in thyroid cancer incidence has been almost universal, it has varied across countries in magnitude [1], as well as by ethnic [2] and other population subgroups [3].

The increase in thyroid cancer incidence is mainly associated with papillary carcinoma, with smaller increases occurring in other types such as follicular carcinoma [3], [4], [5]. While the increase includes both sexes and all age groups, the most rapid increase has occurred among women of reproductive age [6], [7]. The incidence of thyroid cancer is higher in women than men with a consistent ratio of 3∶1, except for in the adolescent and young adult age group where the incidence in females is as much as 5-fold higher [8]. Furthermore, some research shows an increase in predominantly small tumors [9], [10], [11] while other studies indicate increases across all tumor sizes [1], [2], [12].

Ionizing radiation is the best established risk factor for thyroid cancer based on studies of atomic fallout, nuclear accidents, and radiation treatment for benign conditions or previous cancers [13]. While these exposures affect specific groups of people, the population-level risk factors that could account for the widespread increase in thyroid cancer incidence have not yet been identified. One possible explanation for the upward trend is the increasing use of diagnostic technologies allowing the detection of thyroid cancer [10], [11], [14], [15]. Other authors posit a true increase in thyroid cancer incidence due to a widespread increase in a yet unidentified environmental or lifestyle factor(s) [2], [12], [16], [17], [18]

Throughout the world, changes in diet, physical activity and average body weight have tended to occur as countries become more developed, with these changes accounting for as much as 30% of all cancers [19]. This paper critically reviews the literature on the major lifestyle-related risk factors that are most strongly suspected of affecting thyroid cancer rates – body mass index (BMI), diet (including iodine intake), and fish and vegetable consumption. This paper also reviews the major reproductive factors that could account for the higher rates of thyroid cancer among women – pregnancy history, menstrual cycle regularity, menopausal status and the use of prescription hormones.

## Methods

### Search strategy

Although the current paper reviews the evidence on lifestyle and behavioral risk factors, the search strategy was broadly designed to identify all risk factors – lifestyle, behavioral or environmental – for thyroid cancer. The environmental risk factors (diagnostic radiation and endocrine disrupting chemicals) are reviewed in a separate paper (Peterson et al., submitted).

A search for peer-reviewed epidemiological articles was performed using MEDLINE and EMBASE for all articles published between January 1980 and June 2010. The keywords and MeSH terms used for the search were ‘thyroid cancer’ (‘thyroid neoplasm’ for MEDLINE) AND ‘incidence’ OR ‘risk factors’ OR ‘cosmic radiation’ OR ‘electromagnetic radiation’ (‘background radiation’ for MEDLINE) OR ‘ionizing radiation’ OR ‘radiation’ OR ‘radiation dose’(‘radiation effects for MEDLINE) OR ‘radiation exposure’ OR ‘reproduction’ OR ‘estrogen’(EMBASE only) OR ‘hormone’ (EMBASE only) OR ‘parity’(MEDLINE only) OR ‘pregnancy’ (MEDLINE only) OR ‘reproductive history’ (MEDLINE only) OR ‘oral contraceptive agent’ OR ‘iodine’ OR ‘iodine deficiency’ OR ‘diet’ OR ‘body mass’ OR ‘diagnostic test’ (‘diagnostic equipment‘ OR ‘diagnostic techniques’ for MEDLINE) OR ‘endocrine disruptors’ OR ‘pesticides’ OR ‘environmental pollutants’. In addition to this search, other papers were found by manually examining the reference lists of retrieved articles. A search for grey literature was performed using Google Scholar and thyroid-related websites (e.g. professional medical associations and thyroid cancer charities).

### Inclusion and exclusion criteria

In this review, we included English or French language studies that focused on the association of thyroid cancer with each of the following: BMI, female reproductive factors (pregnancy, synthetic hormones, and menstrual factors) and diet, which included iodine intake through fortification or fish consumption. No restrictions were placed on age, sex, geographic region of study and study design. However, studies were required to provide measures of association to be included in the review. If previous pooled studies or meta-analyses were available, we excluded the individual studies of the pooled analysis from our review and examined the pooled results only.

Studies of unique subpopulations were excluded because they are not representative of the general population. These included studies where exposure was from occupation, radioactive iodine-131, treatment for benign thyroid conditions (nodules, goiter, thyroiditis), or the existence of familial or genetic risk factors (MEN2A and MEN2B syndromes; familial adenomatous polyposis (FAP)).

### Review and analysis

Abstracts of papers were reviewed by two authors (EP and PD) to determine their relevance to the inclusion criteria. Any discrepancies between the two reviewers were resolved through discussion and consensus. For abstracts selected for further review, the full papers were retrieved and further reviewed by all authors against the inclusion criteria. The final selection of papers were critiqued using the STROBE checklist (STROBE statement: strengthening the reporting of observational studies in epidemiology, Version 4) as a guide to critically assess studies for selection and measurement bias, confounding, sample size, statistical procedures and generalizability.

Measures of risk (odds ratios, relative risks ratios, hazard ratios) were abstracted from the studies and used to create forest plots. A meta-analysis of the data was not performed due to the heterogeneity of the methods and risk factor definitions across studies. Notable variations in study populations or risk characteristics were, however, indicated on the plots.

## Results

**Figure 1:**
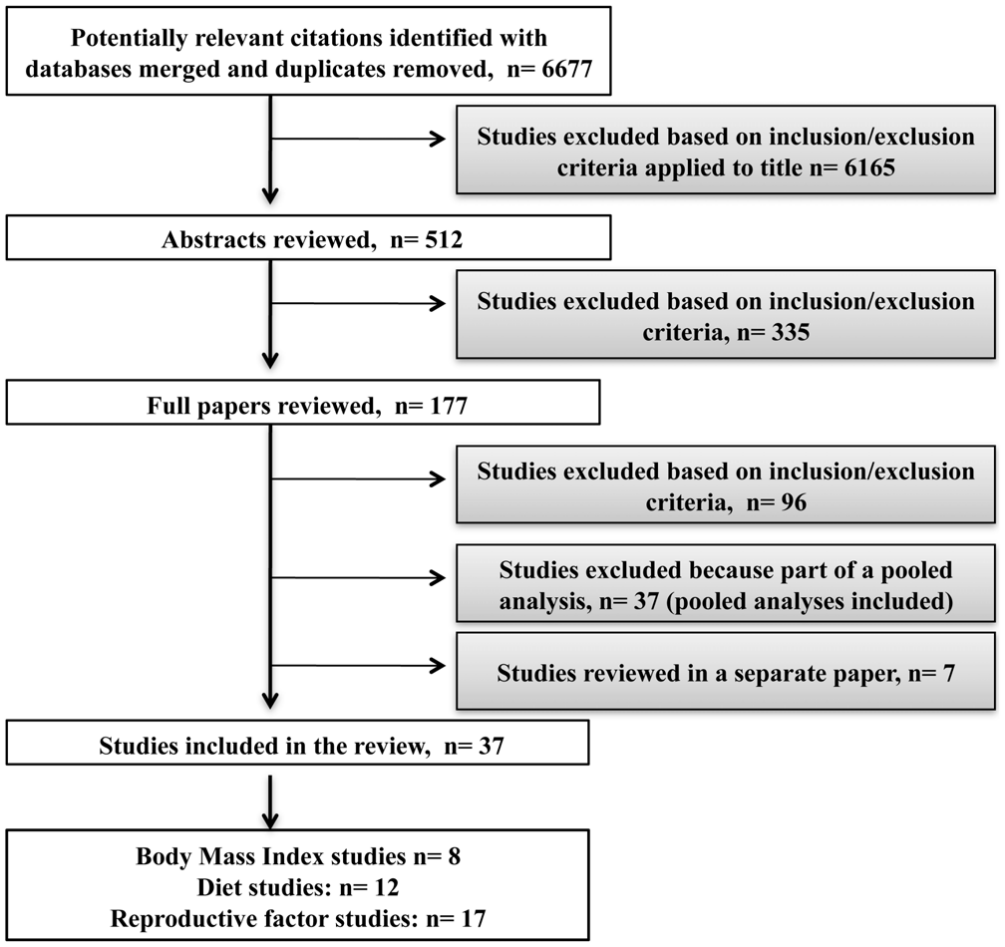
Study selection process. This flowchart includes the study identification process for this paper and studies on environmental risk factors for thyroid cancer that were reviewed in a separate paper.

From an initial list of 6677 potentially relevant studies, 37 studies on BMI, diet and reproductive history were retained for review. No articles were retrieved through the gray literature search. The selection process for the articles is shown in Figure 1 and a range of the risk estimates found for each risk factor is presented in Table 1.

**Table 1:** Range of risk estimates for thyroid cancer for selected risk factors.

Risk factor	Risk estimate range
**Body mass index**	
Males	**1.1–2.3**
Females	**1.0–7.4**
**Diet and iodine intake**	
Iodine supplementation	**0.49–1.6**
Fish consumption	**0.6–2.2**
Cruciferous vegetables	**0.6–1.9**
Non-cruciferous vegetables	**0.71–0.92**
**Reproductive factors**	
Ever pregnant	**0.56–1.1**
Ever parous	**0.73–1.7**
Use of oral contraceptives	**0.6–2.46**
Use of estrogen	**0.6–2.94**
se of hormone replacement therapy	**0.2–1.2**
Irregular menstrual cycle	**1.0–1.9**

### Body mass index

**Figure 2:**
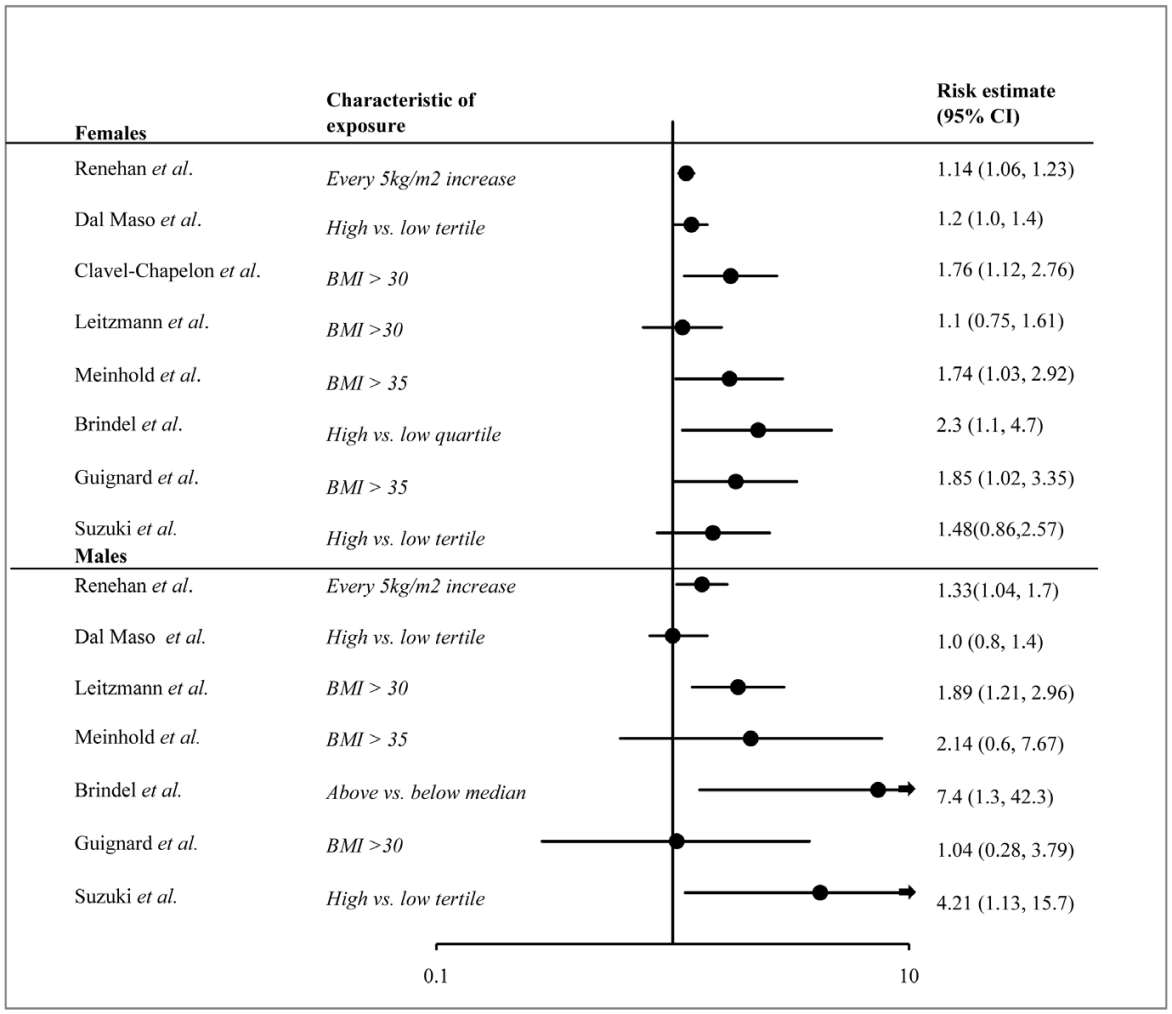
Forest plot of risk estimates for BMI and thyroid cancer for all pathologies of thyroid cancer and all age groups. A meta-analysis of the data was not performed due to the heterogeneity of the methods and risk factor definitions across studies. Notable differences in study characteristics and study samples are indicated on the plot.

We identified 22 studies on BMI that met the inclusion criteria, 5 of which were part of a meta-analysis and 9 were part of a pooled-analysis, which we reviewed. Thus, the 8 studies retained included one meta-analysis, one pooled analysis, three prospective cohort studies and three case-control studies. Summaries of these studies and an assessment of their strengths and weaknesses are presented in Table S1. Most of these studies supported a positive association of BMI with thyroid cancer, with risk estimates ranging from 1.1 to 2.3 in males and 1.0 to 7.4 in females (Figure 2).

A meta-analysis of studies from Europe, Australia and Asia-Pacific looked at the association between BMI and all types of cancer and included five studies that examined thyroid cancer [20]. This analysis showed an increased risk of thyroid cancer in men (RR 1.33, 95%CI 1.04–1.70) and women (RR 1.14, 95%CI 1.06–1.23) for every 5 kg/m^2^ increase in BMI. This meta-analysis included only prospective studies, which establishes a temporal relationship between body mass index and thyroid cancer.

A pooled analysis of twelve case-control studies from the United States, Japan, China and Europe examined BMI and thyroid cancer among 2473 cases and 4323 controls [21]. BMI at the time of diagnosis showed a marginal association with thyroid cancer in females (OR 1.2, 95%CI 1.0–1.4) and no association in males (OR 1.0, 95%CI 0.8–1.4). Since BMI was measured at the time of thyroid cancer diagnosis, a temporal relation between the exposure and outcome could not be determined.

There have been three additional prospective cohort studies on BMI and thyroid cancer published since the meta-analysis. One study on women between the ages of 40 and 65 in France found that for every 5 kg/m^2^ unit increase in BMI there was a 20% elevated risk of thyroid cancer (HR 1.20, 95%CI 1.04–1.38) [22]. A second study of men and women aged 50–71 in the United States found that people who were overweight (BMI = 25–29.9) or obese (BMI≥30) at baseline were at an increased risk of thyroid cancer, with relative risks of 1.27 (95%CI 0.99–1.64) and 1.47 (95%CI 1.03–2.10) respectively [23]. When the analysis of those with a BMI≥30 was stratified by sex, a positive association was observed in males (RR 1.89, 95%CI 1.21–2.96) but not in females (RR 1.10, 95%CI 0.75–1.61). A third study from the United States found that a BMI >35 kg/m^2^ was associated with an increased risk of thyroid cancer in women (HR 1.74, 95%CI 1.03–2.94) but not in men (HR 2.14, 95%CI 0.6–7.67) [24]. Because these three studies were prospective in nature a temporal relationship between thyroid cancer and BMI could be established.

There have also been three additional case-control studies on BMI and thyroid cancer published since the pooled analysis. One study of New Caledonia women found an association between thyroid cancer and a BMI of 30–34.99 (OR 1.92, 95%CI 1.14–3.22) and >35 (OR 1.85, 95%CI 1.02–3.35) [25]. When these measures were stratified by age, women with a BMI of 30–34.99 had an increased risk if they were older than 50 (OR 4.56, 95%CI 1.43–14.54) but not if they were younger than 50 (OR 1.95, 95%CI 0.74–5.17). There was no association between BMI and thyroid cancer found among men in this study. A second case-control study from Japan found that patients with a current BMI of 24–31 were associated with a higher risk of thyroid cancer (OR 1.71, 95%CI 1.06–2.78) when compared to those with a BMI between 16 and 21 [26]. When examined by sex, the risk estimates showed males (OR 4.21, 95%CI 1.13–15.70) being at higher risk than females (OR 1.48, 95%CI 0.86–2.57). A third study in French Polynesia found an increased risk for thyroid cancer in women within the highest quartile of BMI at diagnosis when compared to the lowest (OR 2.3, 95%CI 1.2–4.4) [27]. In males, thyroid cancer risk was increased in those with a BMI at diagnosis above the median compared to those below (OR 7.4, 95%CI 1.3–42.3). Increased BMI at age 18, 30 and 40 was also associated with an increased risk of thyroid cancer in this study.

### Diet

**Figure 3:**
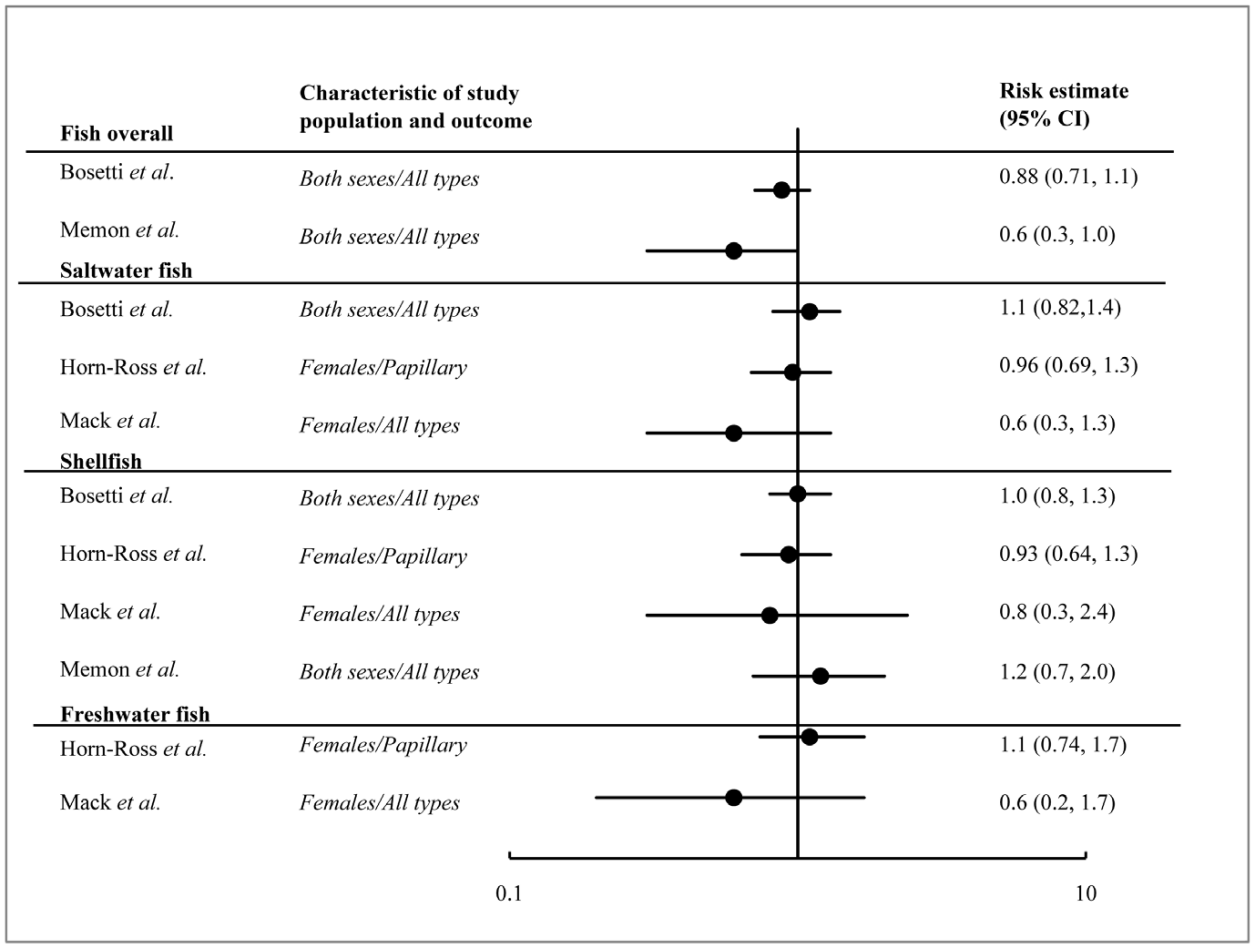
Forest plot of risk estimates for fish consumption and thyroid cancer for all types of fish, saltwater fish, shellfish, and freshwater fish. The plot does not include other types of fish consumption discussed in the paper. A meta-analysis of the data was not performed due to the heterogeneity of the methods and risk factor definitions across studies. Notable differences in study characteristics and study samples are indicated on the plot.

**Figure 4:**
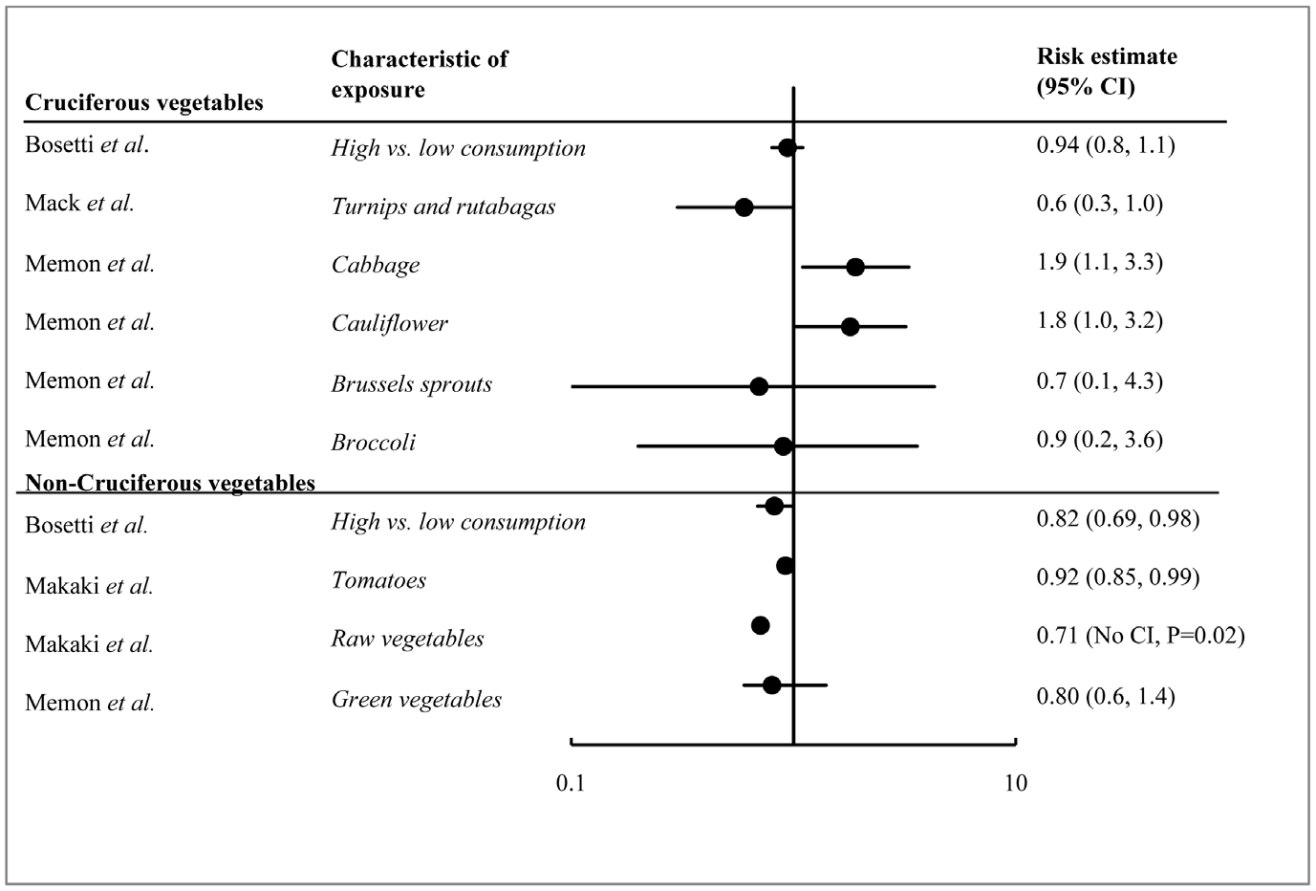
Forest plot of risk estimates for thyroid cancer from consumption of cruciferous and non-cruciferous vegetables. A meta-analysis of the data was not performed due to the heterogeneity of the methods and risk factor definitions across studies. Notable differences in study characteristics and study samples are indicated on the plot.

We identified 21 studies on diet (including iodine intake) and thyroid cancer. Nine were excluded because they were the basis of two pooled analyses, which are included in our analysis. Of the 12 studies retained, most showed a protective (inverse) effect (although generally not statistically significant) with only one showing a positive association of food consumption with thyroid cancer. Summaries of these studies and an assessment of their strengths and weaknesses are presented in Table S2. Risk estimates ranged from 0.49 to 2.2 for various types of dietary exposures (Figures 3 and 4). These studies were inconsistent in the food types that were investigated, which made it difficult to compare results across studies. However, three main categories of food exposures emerged – iodine food fortification, fish consumption and vegetable consumption.

#### Iodine food fortification

The relationship between iodine intake and thyroid cancer is unclear. Research suggests that chronic iodine deficiency is associated with an increased risk of follicular carcinomas, whereas high iodine intake is associated with increased papillary carcinoma [28]. A search for epidemiological studies on iodine intake and thyroid cancer found five studies that met the inclusion criteria; two case-control studies and three ecological studies. The relative risk estimates from these studies ranged from 0.49 to 1.6.

A case-control study in Hawaii included female and male residents above the age of 18 and examined the association between dietary iodine intake and thyroid cancer in 191 cases and 442 controls [29]. When the highest iodine intake quartile was compared to the lowest, a non-significant increased risk for thyroid cancer was found among women (OR 1.6, 95%CI 0.8–3.2) and men (OR 1.3, 95%CI 0.4–3.7).

A second case-control study of 608 women with thyroid cancer and 558 controls between the ages of 20 and 70 in the San Francisco Bay Area examined dietary iodine intake and iodine levels measured in toenail clippings [30]. A significant reduction in the risk of papillary thyroid cancer was seen in the highest iodine intake quintile compared to the lowest (OR 0.49, 95%CI 0.29–0.84); there was no association between dietary iodine and follicular carcinomas. However, significantly lower nail iodine levels were found in cases with follicular cancer than in controls (p = 0.02) but not with papillary cancer.

Three ecological studies each showed an association between iodine intake and papillary carcinoma incidence. One study from Sweden found that the risk of developing papillary carcinoma is lower in regions that are deficient in iodine compared to regions with sufficient iodine (RR 0.80, 95%CI 0.73–0.88) [31]. However, over the study period the age-standardized incidence rates of papillary thyroid cancer increased two-fold in both the iodine deficient and iodine sufficient regions, suggesting that increasing rates are not related to iodine supplementation. A second study found that the age-specific incidence of papillary thyroid cancer was higher in Iceland (a region with high iodine intake) compared to northeast Scotland (a region with low iodine intake), although no statistical analysis was done [32]. A third study from Argentina found a significant increase (p<0.001) in papillary carcinoma in the years after iodine was added to salt, compared to the pre-prophylaxis period [33]. The results of these ecological studies should be interpreted with caution since they did not control for confounding from other known or suspected thyroid cancer risk factors that may have changed over the time period. In addition, the studies that examined two different populations did not account for differences other than iodine intake. Lastly, these studies were done at an ecological level and associations may not necessarily be reflected at the individual level.

#### Iodine through fish consumption

Fish and seafood are one of the main sources of dietary iodine. We found four epidemiological studies on fish consumption and its association with thyroid cancer. One of these was a pooled analysis of case-control studies published between 1980 and 1997 and three were case-control studies published after 1997. The distribution of risk estimates from these studies ranged from 0.6 to 2.2 (Figure 3).

A pooled analysis of thirteen case-control studies included 2497 cases and 4337 controls from the United States, Japan, China and Europe. This study found no association of thyroid cancer among people with the highest level of fish consumption compared to those with the lowest level (OR 0.88, 95%CI 0.71–1.1) [34]. There was a significant decrease in thyroid cancer risk with high fish consumption in regions with endemic goiter (OR 0.65, 95%CI 0.48–0.88) but not in iodine rich regions (OR 1.1, 0.85–1.5). No significant associations were found for saltwater fish consumption or shellfish consumption and thyroid cancer.

There have since been three additional case control studies on fish consumption and thyroid cancer. A study of women in the San Francisco Bay Area found no association between papillary thyroid cancer and any type of fish consumption – shellfish, saltwater fish, freshwater fish or canned tuna [30]. A second study from Kuwait found a protective effect with the consumption of more than 2 servings of fresh fish per week (OR 0.5, 95%CI 0.4–0.7) but an increased risk of thyroid cancer with the consumption of processed, canned or frozen fish (OR 2.2, 95%CI 1.6–3.0)[35]. The authors suggested that the elevated risk associated with processed fish may be related to higher iodine levels in processed fish or to altered iodine uptake due to additives or processing. No association with thyroid cancer was found with total fish consumption or shellfish consumption. A third study of women in Los Angeles County found no association between any amount of fish consumption (shellfish, saltwater fish and freshwater fish) consumption and thyroid cancer risk [36].

#### Vegetable consumption

Cruciferous vegetables (e.g. cabbage, Brussel sprouts, broccoli, cauliflower, radish, turnip) contain thioglucosides, which can induce thyroid cancer in laboratory animals [37]. We reviewed four epidemiological studies on cruciferous and non-cruciferous vegetable intake and thyroid cancer. One was a pooled analysis of studies published between 1980 and 1997 and three were case-control studies that looked at multiple dietary risk factors, including vegetable intake. Risk estimates for cruciferous vegetable consumption ranged from 0.6 to 1.9, and for non-cruciferous vegetables ranged from 0.71 to 0.92 (Figure 4).

A pooled analysis of 11 case-control studies from the United States, Asia and Europe considered vegetable intake in 2241 cases and 3716 controls [38]. This analysis found no significant association of thyroid cancer among people who consumed the highest amount of total cruciferous vegetables compared to those with the lowest consumption levels (OR 0.94, 95%CI 0.80–1.10). There was, however, a decrease in thyroid cancer risk among those who consumed the highest amounts of non-cruciferous vegetables compared to those with the lowest consumption levels (OR 0.82, 95%CI 0.69–0.98).

There have since been three additional case-control studies published after the pooled analysis. One from Kuwait found an increased risk of thyroid cancer with the consumption of cabbage (OR 1.9, 95%CI 1.1–3.3), a marginal increase in risk with the consumption of cauliflower (OR 1.8, 95%CI 1.0–3.2), and no association with the consumption of Brussels sprouts, broccoli or green vegetables [35]. A second study of women in Los Angeles found a decreased risk of thyroid cancer with the consumption of cruciferous vegetables (turnips and rutabagas) (OR 0.6, 95%CI 0.3–1.0) [36]. A third study from Greece found a decreased risk with the consumption of a select group of non-cruciferous raw vegetables (OR 0.71, P = 0.02) and raw tomatoes (0.92, 95%CI 0.85–0.99) [39].

**Other dietary items:** We reviewed three other case-control studies that looked at the relationship between thyroid cancer and food items or groups other than iodine, fish or vegetables. Because these studies each examined different dietary factors, no consistent patterns can be described. One study from northern Italy found an increased risk of thyroid cancer with high refined cereal intake (OR 2.0, 95%CI 1.4–2.9) and a marginal association with low beta-carotene intake (OR 1.4, 95%CI 1.0–1.9) [40]. The authors suggested that these measures are likely indicative of an overall poor diet. A second study of women in the San Francisco Bay Area found that increased consumption of total phytoestrogens (mainly through soy consumption) was associated with a decreased risk of thyroid cancer (OR 0.62, 95%CI 0.39–0.99) [41]. Phytoestrogens are estrogenic compounds found in plant foods, such as soy, that can bind with estrogen receptors. A third study from Greece conducted a factor analysis of various combinations of food items to determine their association with thyroid cancer [39]. A combination of raw vegetables typically found in a Greek salad decreased the risk of thyroid cancer. A decreased risk for thyroid cancer was also found with the consumption of fruits (OR 0.68, p = 0.01) and mixed vegetables and fruits (OR 0.73, p = 0.04). An increased risk for follicular carcinoma was associated with the consumption of a combination of fish and cooked vegetables (OR 2.79, p = 0.02).

### Reproductive factors

We identified 29 studies on reproductive factors that covered the topics of pregnancy, menstrual cycle, menopause and use of prescription hormones. Twelve studies were excluded because they were part of pooled analyses, which are included in our review. Thus, of the 17 studies we reviewed there were 2 pooled analyses, 6 cohort studies and 9 case-control studies. These studies are discussed in detail in the Text S1 and Table S3.

Among these studies, the common reproductive factors that were assessed were: ever being pregnant, ever being parous, number of pregnancies and live births, use of prescription hormones (including oral contraceptives (OC), estrogen and hormone replacement therapy), menstrual cycle regularity, and menopausal status. Individual risk estimates showed wide variation – pregnancy (0.56–1.1), number of live births (0.73–1.7), OC use (0.6–2.46), estrogen use (0.6–2.94), HRT use (0.2–1.2), and menstrual cycle irregularity (1.0–1.9) – and none of these risk factors were consistently associated with a change in risk of thyroid cancer (see Figures S1, S2, and S3).

## Discussion

Numerous epidemiological studies have looked at risk factors for thyroid cancer in order to explain the increasing rates of this cancer throughout the world and specifically among women. In this report, we critically reviewed the risk factors that are associated with diet and nutrition and those related to a woman's reproductive history. No one factor was consistently associated with a strong increase in thyroid cancer risk, although a higher BMI did show mostly positive associations. The studies reviewed here were performed in different countries but there was no clear directionality in thyroid cancer risk with ethnicity of the study population.

While some of the reviewed risk factors were the subject of a recent review by Dal Maso and colleagues [42], our study updates and expands on that review and highlights the limitations of available research. The most common limitations across the studies we reviewed were i) a lack of statistical adjustment for other putative thyroid cancer risk factors, ii) small numbers of thyroid cancer cases among study populations, which lowered statistical power, iii) low accuracy or reliability of exposure information due to self-reported data, iv) few prospective studies to adequately assess temporality of exposure to a risk factor and development of thyroid cancer and v) a lack of simultaneous consideration of multiple risk factors in the assessment of thyroid cancer risk.

### Body mass index (BMI)

Several studies showed that a higher BMI is weakly associated with an elevated risk of thyroid cancer. No clear trend was observed between study population or thyroid tumor type and the association between BMI and thyroid cancer. However, one study did find a positive association of BMI with papillary, follicular and anaplastic thyroid cancers but not with medullary thyroid cancer [23]. Half of the studies reviewed here were prospective cohort studies suggesting that a higher BMI preceded a diagnosis of thyroid cancer.

Worldwide, obesity rates have been increasing over the last 30 years. In Canada, for example, the average BMI for a typical forty-five year old male or female have increased by approximately 2kg/m^2^ between 1981 and 2009 [43]. It is possible that the increasing trend in obesity (and thus rising BMI) in the general population may be an important factor contributing to the rising thyroid cancer incidence. However, the inconsistent and weak association in the studies we reviewed cannot fully account for the large observed increases in thyroid cancer incidence.

A common weakness of studies on BMI and thyroid cancer is that the height and weight information used to calculate BMI is almost always self-reported, which may lead to inaccurate or misclassified exposure status. A systematic review comparing self-reported height and weight to actual measurements found that people tended to underestimate their weight and overestimate their height leading to an underestimation of BMI [44]. Whether this misclassification is different for each BMI category is unknown and therefore its impact on over or underestimating risk cannot be determined.

The association between BMI and thyroid cancer may be confounded by multiple factors including an increased risk of diabetes, although there has been one study showing this is not the case [23]. Also, it is unknown whether physical activity has an impact on thyroid cancer, with one study finding a positive association [45] and a second finding no association [23]. Other researchers suggest that the association between BMI and thyroid cancer is due to detection bias because overweight and obese people tend to see the doctor more often where they may have examinations of the thyroid, but other research has argued against this [25]. Based on the current evidence, it is unclear if any of these factors can account for the weak association found between BMI and thyroid cancer.

An increased BMI has been associated with many other tumors in addition to thyroid cancer suggesting a general biological mechanism between obesity and cancer [20]. The biological processes linking high BMI to cancer risk have not yet been clearly identified, but the potential mechanisms include insulin and insulin-like growth factor I, sex steroids, or adipokines acting on the thyroid to stimulate cell proliferation and suppress apoptosis [46].

### Diet

#### Iodine food fortification

Iodine fortification of food has been increasing whereby 70% of the world's population consumed iodized salt in 2000 compared to 20% in 1990 [47]. There has been research to suggest that chronic iodine deficiency is associated with an increased risk of follicular carcinomas, whereas high iodine intake is associated with increased papillary carcinoma [28]. Our review of the published studies shows that the impact of iodine intake on the risk of papillary thyroid cancer is unclear. Two case-control studies showed that papillary thyroid cancer risk did not significantly increase with increased iodine intake and one study showed a significant decrease in risk. It should be noted that iodine intake categories among studies were not comparable, which makes it difficult to discriminate between moderate and high iodine intake. The ecological studies we reviewed all suggested an increase in papillary cancer with increasing iodine intake, although there are multiple confounding factors that could account for these observations, including other environmental risk factors, changes in histological definitions or differences in medical practices.

There is evidence that changes in iodine intake can impact the ratio of papillary to follicular thyroid cancers. Population studies have found that iodine supplementation increases the proportion of papillary thyroid cancer while iodine deficiency increases the proportion of follicular thyroid cancer in the population exposed to such changes [48]. It has been suggested that iodine intake changes the ratio of papillary to follicular cancers, without actually changing the overall incidence of thyroid cancer [49]. In our analysis, the combination of inconsistent results from case-control studies and methodological weaknesses among ecological studies supports the conclusion that worldwide changes over time in iodine intake may not be an important factor in increasing thyroid cancer incidence rates.

#### Other dietary factors

It is possible that other dietary factors may affect thyroid cancer risk. Fish and seafood are one of the main sources of dietary iodine, and their consumption occurs at high levels in geographic areas where the incidence of papillary thyroid cancer is also highest, such as Iceland, Norway and Hawaii. While the consumption of fruits and vegetables can decrease the risk of certain types of cancer[19], cruciferous vegetables (e.g. cabbage, Brussels sprouts, broccoli, cauliflower, radish, turnip) are considered to be goitrogenic since they can block iodine uptake and a large intake of them can increase thyroid stimulating hormone (TSH).

In our analysis, the consumption of fish had no consistent association with thyroid cancer in the studies we reviewed, although a pooled analysis suggested a protective effect in geographic regions with endemic goiter. The only consistent relationship we found is that diets high in non-cruciferous raw vegetables result in a slightly reduced risk of thyroid cancer, suggesting a possible protective effect. On the other hand, diets high in cruciferous vegetables were not associated with an increased risk, as has been confirmed by animal studies which examined the role of goitrogens on the thyroid [37]. As for other dietary factors, the research is inconsistent in terms of the factors studied and the magnitude of the risks identified, making it difficult to identify specific associations. Based on studies we reviewed, diet does not appear to be a factor that could explain the increased thyroid cancer incidence observed worldwide.

All of the studies on diet and thyroid cancer were case-control studies, which used retrospective interviews to determine exposure and thus had the possibility of recall bias. Prospective cohort data could help overcome this methodological barrier. Also, selection bias was another major limitation as cases were often recruited through methods that were different from the controls. In addition, the association between thyroid cancer and diet could not be adequately examined with respect to the impact of gender because most studies were restricted to females only or to both sexes combined. Lastly, it was difficult to make comparisons between studies as they often examined different dietary components and items, or differed in quantity and frequency of consumption. Future studies on diet and thyroid cancer will need to overcome these issues.

### Reproductive factors

The risk of thyroid cancer in women increases at the time of puberty and declines after menopause whereas men have a steady increase in risk throughout their lifetime [13], providing support to the notion that hormonal factors are involved in some thyroid cancers. It has been hypothesized that estrogen increases the levels of TSH in the body, in turn increasing thyroid growth [13]. In fact, estrogen receptors are highly expressed in human thyroid neoplasms [13] and estrogen, along with pregnancy and oral contraceptives use, are all associated with elevated serum thyroxin and triiodothyronine levels, which might induce high cell turnover[50]. The highest levels of serum TSH, even within the normal range, are associated with a subsequent diagnosis of thyroid cancer in individuals with thyroid abnormalities[51]. In our review, the epidemiologic evidence showed only weak and equivocal associations between the major pregnancy, hormonal and menstrual cycle factors and thyroid cancer risk.

### Conclusion

Given that cancer is sometimes the result of multiple genetic, environmental and lifestyle factors working cumulatively to increase risk, the weak individual effect sizes identified in this review suggest a synergistic process. For example, iodine deficiency can promote the effects of radiation on the thyroid gland [52]. Levels of TSH, which change as a result of exposure to various reproductive factors and environmental chemicals, can encourage pre-existing tumours to grow and develop into cancer [53]. In addition to this review, we have also performed a review of the environmental factors which may contribute to this synergistic process (Peterson E. et al, submitted).

Future research should collect population-level exposure data through better risk factor surveillance. More research is also needed to explain the differences in thyroid cancer among women and men. This could help identify and support prevention efforts that aim to reduce the burden of thyroid cancer.

## Supporting Information

Text S1 **Female reproductive factors associated with thyroid cancer.**
(DOC)

Figure S1 **Forest plot of risk estimates for major pregnancy factors and thyroid cancer.** A meta-analysis of the data was not performed due to the heterogeneity of the methods and risk factor definitions across studies. Notable differences in study characteristics and study samples are indicated on the plot.
(TIF)

Figure S2 **Forest plot of risk estimates for major hormonal factors and thyroid cancer.** A meta-analysis of the data was not performed due to the heterogeneity of the methods and risk factor definitions across studies. Notable differences in study characteristics and study samples are indicated on the plot.
(TIF)

Figure S3 **Forest plot of risk estimates for menstural cycle irregularity and thyroid cancer.** A meta-analysis of the data was not performed due to the heterogeneity of the methods and risk factor definitions across studies. Notable differences in study characteristics and study samples are indicated on the plot.
(TIF)

Table S1 **Summary of reviewed studies on BMI (body mass index) and thyroid cancer.**
(DOCX)

Table S2 **Summary of reviewed studies on diet and thyroid cancer.**
(DOCX)

Table S3 **Summary of reviewed reproductive factor studies and thyroid cancer.**
(DOCX)
