# Supplementary material for: BMI, Diet and Female Reproductive Factors as Risks for Thyroid Cancer: A Systematic Review
Source: PLoS One. 2012 Jan 19;7(1):e29177. doi: 10.1371/journal.pone.0029177 (PMC3261873; doi:10.1371/journal.pone.0029177)
Supplement: Text S1 — Female reproductive factors associated with thyroid cancer. (DOC) [file pone.0029177.s001.doc]

**Female reproductive factors associated with thyroid cancer**

**Introduction**

Thyroid cancer incidence rates have been increasing throughout the world, with females having a higher rate than males [1]. Here, we critically review the literature on the major reproductive factors that could account for the higher rates of thyroid cancer among women – pregnancy history, menstrual cycle regularity, menopausal status and the use of prescription hormones.

**Methods**

The search strategy, inclusion and exclusion criteria, and the review and analysis process are described in the main article.

**Results**

We identified 29 studies on reproductive factors that covered the topics of pregnancy, menstrual cycle, menopause and use of prescription hormones. Twelve studies were excluded because they were part of a pooled analysis. In the 17 studies we reviewed, there were 2 pooled analyses, 6 cohort studies and 9 case-control studies (Table S3).

Generally, a history of pregnancy and number of live births (Figure S1), use of oral contraceptives (OCs), estrogen or hormone replacement therapy (HRT) (Figure S2), and menstrual cycle irregularity (Figure S3) were weakly and inconsistently associated with thyroid cancer risk. Risk estimates showed wide variation: pregnancy (0.56-1.1), number of live births (0.73-1.7), OC use (0.6-2.46), estrogen use (0.6-2.94), HRT use (0.2-1.2), and menstrual cycle irregularity (1.0-1.9).

Two pooled analyses of case-control studies [2,3] have been published. One analysis of 13 studies published between 1980 and 1997 from the United States, Asia, and Europe found an elevated, but non-significant, risk of thyroid cancer associated with the use of oral contraceptives (OR 1.2, 95%CI 1.0-1.4) [2]. There was no association with duration of OC use, age at first use, use before first birth, or geographic regions. Interestingly, the risk for current users of OCs, although not significantly elevated, declined after 10 years of stopping OC use. This analysis also examined HRT and fertility drugs but found no association with thyroid cancer for both factors. Lactation suppression treatment, on the other hand, was the only factor significantly associated with higher risk of thyroid cancer (OR 1.5, 95%CI 1.1-2.1) and was highest for those at a younger age at diagnosis.

A separate pooled analysis of 14 studies published between 1980 and 1997 from the United States, Asia and Europe found that artificial menopause (for example, removal of the ovaries) elevated the risk of thyroid cancer (OR 1.8, 95% CI1.4-2.4) [3]. There was a small and non-significant association between late menarche and thyroid cancer risk (OR 1.04, 95% CI 1.0-1.1). Furthermore, miscarriage at first pregnancy was the only pregnancy-related factor that was significantly associated with higher risk (OR 1.8, 95% CI 1.2-2.6).

We identified 6 cohort studies on reproductive factors published since the pooled analyses. A prospective cohort study of over 89,000 Canadian women enrolled in the National Breast Screening Study collected information on menstrual and reproductive history through a questionnaire administered at enrollment [4]. Women were followed for 20 years and incident cases of thyroid cancer were ascertained from a population based registry. This study found no evidence of altered thyroid cancer risk associated with parity, age at first live birth, age at menarche, menopausal status at time of diagnosis, use or OCs and use of HRT.

A record-linkage cohort study from Sweden obtained birth information, including number of pregnancies and births of twins, from the Civil Birth Registry [5]. These birth cases were then compared to the thyroid cancer cases from the Swedish Cancer Registry. There was no significant association with number of pregancies, age of first birth or twinning on risk of thyroid cancer.

In a large case-cohort analysis of 54,362 women from Danish fertility clinics [6], use of the fertility drug clomiphene was linked to elevated thyroid cancer risk in all women, and especially in those who were parous. A history of progesterone use was also associated with risk but with large uncertainty about the risk estimate, particularly since the analysis was based on 29 thyroid cancer cases of whom few used progesterone.

A cohort study of female textile workers in Shanghai showed that a history of OC use was not associated with thyroid cancer in this population [7]. However, the study was not specific to thyroid cancer (9 cancers types were examined) and therefore did not control for all confounders for thyroid cancer. A second cohort study from Shanghai that also looked at several cancers found no association with OC use and thyroid cancer [8].

Investigators with the Japan Collaborative Cohort Study enrolled almost 38,000 women and collected information on menstrual and reproductive factors as well as hormone usage through a questionnaire administered at enrollment [9]. Women were followed for at least 5 years and incidence of thyroid cancer was ascertained from local cancer registries. The authors found no association between thyroid cancer risk and age at menarche, age at menopause parity, being pregnant, age at first birth and hormone use.

We identified 9 case control studies on reproductive factors that were either published immediately before the pooled analyses, and thus were not included, or published afterwards. An analysis of women in Washington State found no association of papillary thyroid cancer with OC or HRT use among women aged 45-64 years [10]. For women younger than age 45, OC use was associated with a reduced risk of papillary thyroid cancer (OR 0.6, 95% CI 0.4-0.9) although the amount of estrogen and duration of use did not affect overall risk.

A case-control study from Los Angeles included women aged 15-54 at the time of a thyroid cancer diagnosis and found no association between thyroid cancer and several reproductive factors including age at menarche, irregular menstruation, ever being pregnant, ever having had a miscarriage, number of pregnancies, age at first live birth, ever breastfeeding and use of most reproductive hormones [11]. The authors did, however, find an increase in thyroid cancer risk among women who had had a hysterectomy (OR 1.9, 95% CI 1.0-3.8), and more specifically with women who had had a hysterectomy with complete oophorectomy (OR 6.5, CI 1.1-38.1). The authors also found a weak association with thyroid cancer risk and the use of lactation suppressants (ever use: OR 1.7, 95% CI 0.9-3.4) with the risk increasing with the number of pregnancies in which lactation suppressants were used (p-value trend = 0.03).

A later analysis of women from the Washington State case control study showed that among women 45-64 years of age, there was no association of papillary thyroid cancer with the number of live births, age of the mother at first live birth, her age at last live birth, or breastfeeding history [12]. Among women younger than 45, having had more 2 or more live births in the past 5 years compared to nulliparous women (OR 2.1, 95% CI 0.9-4.6), higher duration of lifetime lactation and decreased time since last lactation were associated with an increased risk of thyroid cancer.

A case-control study from Kuwait identified thyroid cancer cases from a national cancer registry and found no association between thyroid cancer and several reproductive factors including age at menarche, ever being pregnant, number of pregnancies, menopausal status and use of female hormones, OCs, infertility drugs, lactation suppressants or HRT [13]. This study did show a significant increase in thyroid cancer risk with an older age at last pregnancy (p-value trend <0.05), an increase in risk in women with a history of thyroiditis (OR 10.2, 95% CI 2.3-44.8) and a decrease in risk with an increasing number of miscarriages (p-value trend <0.05).

A case-control study from the San Francisco Bay Area [14] was the only study we identified that showed age at menarche as being associated with thyroid cancer risk, with an age of menarche younger than 12 (OR 1.5, 95% CI 1.1-2.3) or older than 15 (OR 1.5, 95% CI 0.98-2.3) having a higher risk relative to an age of menarche of 12. There was also a weak association with parity (OR 1.4, 95% CI 0.98-2.1), especially for women who had two live births (OR 1.7, 95% CI 1.1-2.7) compared to nulliparous women. An older age at first and at last full-term pregnancy was also associated with higher thyroid cancer risk. Use of OCs was associated with a decrease in thyroid cancer risk (OR 0.73, CI 0.52-0.97) while use of HRT was not associated. There was no association between thyroid cancer risk and menopausal status or a history of miscarriage.

A study of women in Italy aged 45-79 who were identified through several case control studies looked at the effect of HRT use on the risk of numerous cancers [15]. The authors found that HRT use was not associated with thyroid cancer risk, regardless of duration of use (ever, <2 years, or >2 years).

A matched case-control study of women diagnosed with thyroid cancer in Yugoslavia found that spontaneous abortion (OR 1.98, 95% CI 1.03-3.50) and OC use (OR 2.34, 95% CI 1.31-4.18) were associated with elevated risk of thyroid cancer when assessed by multivariate regression analysis [16]. However, none of these factors were associated with thyroid cancer when adjusted for other factors, including residence in an area with endemic goiter, exposure to radioactive iodine or history of thyroid or other endocrine disorders.

A matched case-control analysis of women over the age of 18 from New Caledonia in the South Pacific [17] found that the risk of thyroid cancer was significantly associated with irregular menstruation for all ages (OR 1.9, 95% CI 1.2-3.2), and especially for women younger than 45 years (OR 3.6, 95% CI 1.7-7.4) and those of Melanesian ethnicity (OR 3.0, 95%CI 1.3-6.7). In addition, having more than 8 live births (OR 2.2, 95% CI 1.1-4.3, compared to nulliparous) and ever having had an abortion (OR 3.1, 95% CI 1.5-6.2) were linked to higher risk. There appeared to be a dose-response trend with the number of live births but the association was only significant for more than 8 births. In this population, no association was found with OC or HRT use, age at menarche, hysterectomy or a history of miscarriage.

A case-control study from French Polynesia [18] found that thyroid cancer risk is higher among women with a higher number of live births (p-value trend = 0.03). Thyroid cancer risk was also higher among women who underwent artificial menopause (OR 5.4, 95% CI 1.7-12.0) compared to women who are still menstruating; there was no association for women who had undergone natural menopause compared to women still menstruating. There was no association between thyroid cancer and age at menarche, ever being pregnant and history of miscarriage.

**Discussion**

The risk of thyroid cancer in women increases at the time of puberty and declines after menopause whereas men have a steady increase in risk throughout their lifetime [19], providing support to the notion that hormonal factors are involved in some thyroid cancers. It has been hypothesized that estrogen increases the levels of thyroid stimulating hormone (TSH) in the body, in turn increasing thyroid growth [19]. In fact, estrogen receptors are highly expressed in human thyroid neoplasms [19] and estrogen, along with pregnancy and oral contraceptives use, are all associated with elevated serum thyroxin and triiodothyronine levels, which might induce high cell turnover[20]. The highest levels of serum TSH, even within the normal range, are associated with a subsequent diagnosis of thyroid cancer in individuals with thyroid abnormalities[21]. In our review, the epidemiologic evidence showed only weak and equivocal associations between the major pregnancy, hormonal and menstrual cycle factors and thyroid cancer risk. The fact that there were inconsistencies in terms of types of factors studied, how risk factors were categorized, and magnitude of risk make it difficult to identify associations.

There were, however, a number of trends that occur throughout the studies to suggest a small role of hormones in stimulating thyroid activity. First, there is a small increased risk among women with current menopause compared to women who had menopause 10 years earlier. Second, there is inconsistent risk with miscarriages, possibly as a result of susceptibility to miscarriage from benign thyroid disease, which serves as a proxy measure. Third, late menarche (after age 15) slightly increases future risk of thyroid cancer. Fourth, recent pregnancy (in the past 2 years) slightly increases risk of papillary cancer, especially for those who have at least 2 live births within 5 years. This may be due to the increased production of TSH during pregnancy. Finally, fertility drugs used to stimulate reproductive hormones and ovulation have also been linked to increased risk; women who take progesterone and clomiphene are at greater risk of developing thyroid cancer [6].

**References**

1. Kilfoy BA, Devesa SS, Ward MH, Zhang Y, Rosenberg PS, et al. (2009) Gender is an age-specific effect modifier for papillary cancers of the thyroid gland. Cancer Epidemiology, Biomarkers & Prevention 18: 1092-1100.

2. La Vecchia C, Ron E, Franceschi S, Dal Maso L, Mark SD, et al. (1999) A pooled analysis of case-control studies of thyroid cancer. III. Oral contraceptives, menopausal replacement therapy and other female hormones. Cancer Causes & Control 10: 157-166.

3. Negri E, Dal Maso L, Ron E, La Vecchia C, Mark SD, et al. (1999) A pooled analysis of case-control studies of thyroid cancer. II. Menstrual and reproductive factors. Cancer Causes & Control 10: 143-155.

4. Navarro Silvera SA, Miller AB, Rohan TE (2005) Risk factors for thyroid cancer: a prospective cohort study. International Journal of Cancer 116: 433-438.

5. Neale RE, Darlington S, Murphy MFG, Silcocks PBS, Purdie DM, et al. (2005) The effects of twins, parity and age at first birth on cancer risk in Swedish women. Twin Research and Human Genetics 8: 156-162.

6. Hannibal CG, Jensen A, Sharif H, Kjaer SK (2008) Risk of thyroid cancer after exposure to fertility drugs: results from a large Danish cohort study. Human Reproduction 23: 451-456.

7. Rosenblatt KA, Gao DL, Ray RM, Nelson ZC, Wernli KJ, et al. (2009) Oral contraceptives and the risk of all cancers combined and site-specific cancers in Shanghai. Cancer Causes & Control 20: 27-34.

8. Dorjgochoo T, Shu XO, Li HL, Qian HZ, Yang G, et al. (2009) Use of oral contraceptives, intrauterine devices and tubal sterilization and cancer risk in a large prospective study, from 1996 to 2006. International Journal of Cancer 124: 2442-2449.

9. Pham TM, Fujino Y, Mikami H, Okamoto N, Hoshiyama Y, et al. (2009) Reproductive and menstrual factors and thyroid cancer among Japanese women: The Japan collaborative cohort study. Journal of Women's Health 18: 331-335.

10. Rossing MA, Voigt LF, Wicklund KG, Williams M, Daling JR (1998) Use of exogenous hormones and risk of papillary thyroid cancer (Washington, United States). Cancer Causes & Control 9: 341-349.

11. Mack WJ, Preston-Martin S, Bernstein L, Qian D, Xiang M (1999) Reproductive and hormonal risk factors for thyroid cancer in Los Angeles County females. Cancer Epidemiology, Biomarkers & Prevention 8: 991-997.

12. Rossing MA, Voigt LF, Wicklund KG, Daling JR (2000) Reproductive factors and risk of papillary thyroid cancer in women. American Journal of Epidemiology 151: 765-772.

13. Memon A, Darif M, Al-Saleh K, Suresh A (2002) Epidemiology of reproductive and hormonal factors in thyroid cancer: Evidence from a case-control study in the Middle East. International Journal of Cancer 97: 82-89.

14. Sakoda LC, Horn-Ross PL (2002) Reproductive and menstrual history and papillary thyroid cancer risk: the San Francisco Bay Area thyroid cancer study. Cancer Epidemiology, Biomarkers & Prevention 11: 51-57.

15. Fernandez E, Gallus S, Bosetti C, Franceschi S, Negri E, et al. (2003) Hormone replacement therapy and cancer risk: A systematic analysis from a network of case-control studies. International Journal of Cancer 105: 408-412.

16. Zivaljevic V, Vlajinac H, Jankovic R, Marinkovic J, Dzodic R, et al. (2003) Case-control study of female thyroid cancer - Menstrual, reproductive and hormonal factors. European Journal of Cancer Prevention 12: 63-66.

17. Truong T, Orsi L, Dubourdieu D, Rougier Y, Hemon D, et al. (2005) Role of goiter and of menstrual and reproductive factors in thyroid cancer: a population-based case-control study in New Caledonia (South Pacific), a very high incidence area. American Journal of Epidemiology 161: 1056-1065.

18. Brindel P, Doyon F, Rachedi F, Boissin JL, Sebbag J, et al. (2008) Menstrual and reproductive factors in the risk of differentiated thyroid carcinoma in native women in French Polynesia: a population-based case-control study. American Journal of Epidemiology 167: 219-229.

19. Schottenfeld D, Fraumeni Jr JF, editors (2006) Cancer Epidemiology & Prevention. Third Edition ed: Oxford University Press.

20. Adami HO, Hunter D, Trichopoulos D, editors (2008) Textbook of Cancer Epidemiology. USA: Oxford University Press.

21. Haymart MR, Repplinger DJ, Leverson GE, Elson DF, Sippel RS, et al. (2008) Higher Serum TSH Level in Thyroid Nodule Patients is Associated with Greater Risks of Differentiated Thyroid Cancer and Advanced Tumor Stage. Journal of Clinical Endocrinology and Metabolism 93: 809-814.
