# Supplementary figures and images for: BMI, Diet and Female Reproductive Factors as Risks for Thyroid Cancer: A Systematic Review
Source: PLoS One. 2012 Jan 19;7(1):e29177. doi: 10.1371/journal.pone.0029177 (PMC3261873; doi:10.1371/journal.pone.0029177)

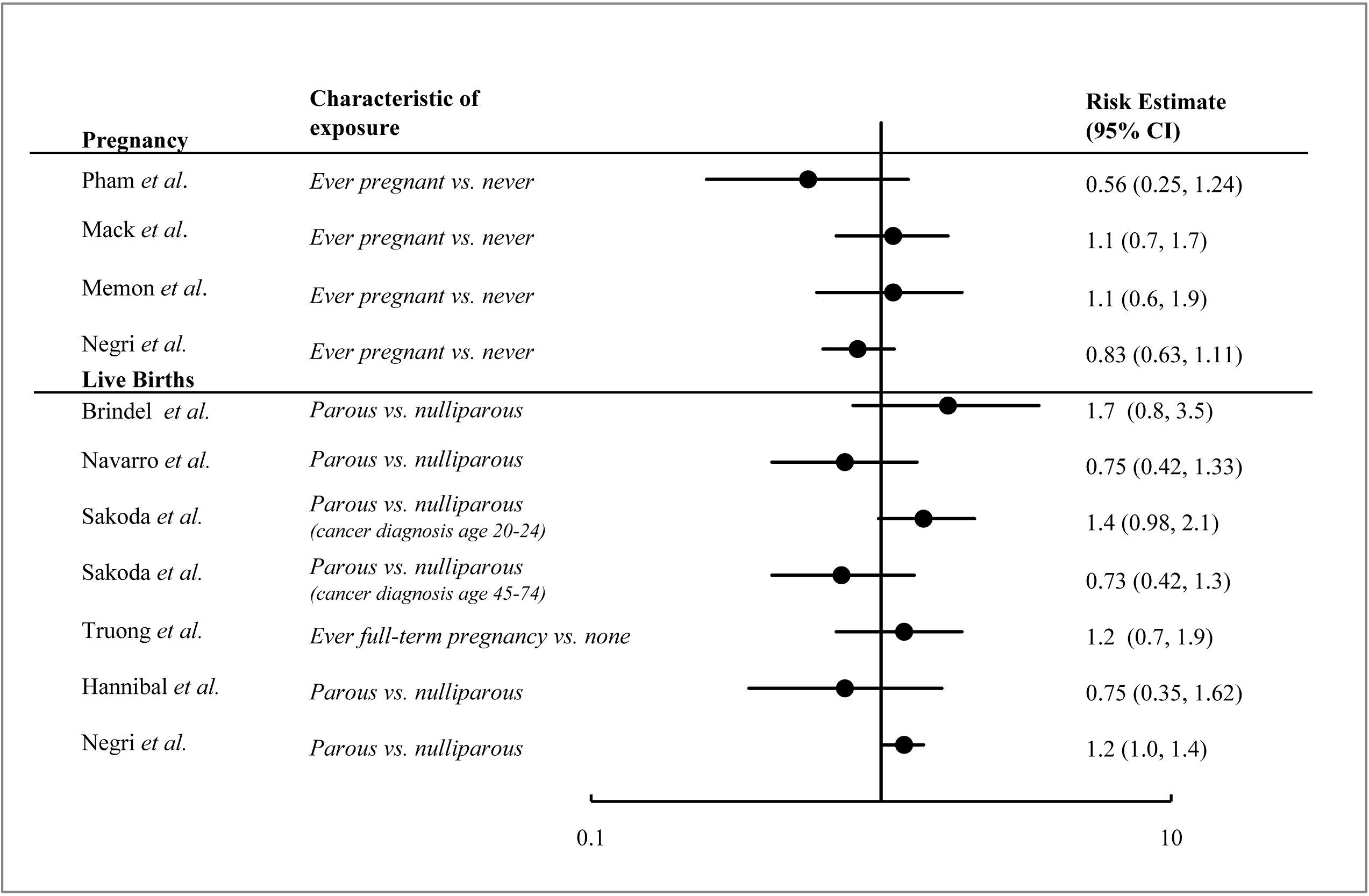

Supplement: Figure S1 — Forest plot of risk estimates for major pregnancy factors and thyroid cancer. A meta-analysis of the data was not performed due to the heterogeneity of the methods and risk factor definitions across studies. Notable differences in study characteristics and study samples are indicated on the plot. (TIF) [file pone.0029177.s002.tif]

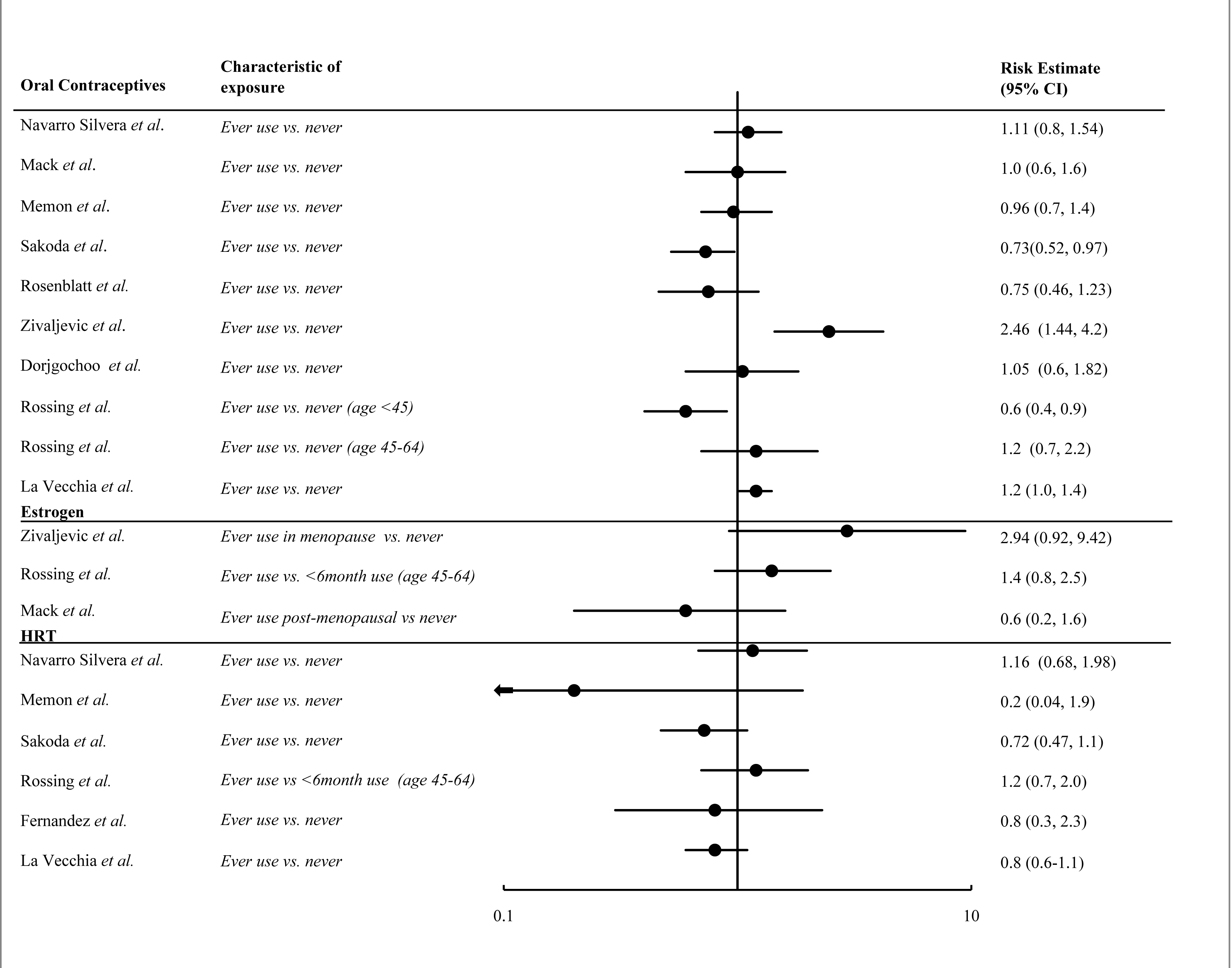

Supplement: Figure S2 — Forest plot of risk estimates for major hormonal factors and thyroid cancer. A meta-analysis of the data was not performed due to the heterogeneity of the methods and risk factor definitions across studies. Notable differences in study characteristics and study samples are indicated on the plot. (TIF) [file pone.0029177.s003.tif]

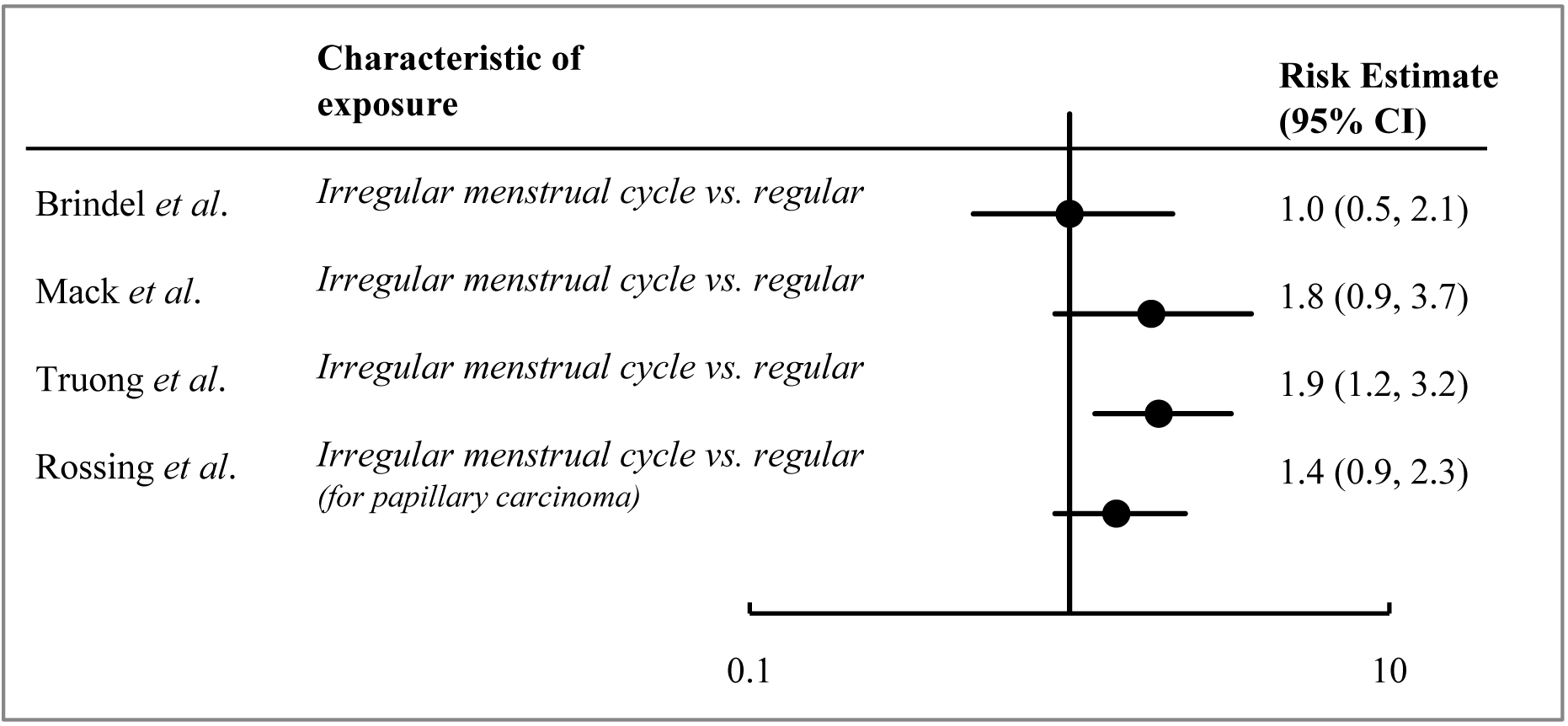

Supplement: Figure S3 — Forest plot of risk estimates for menstural cycle irregularity and thyroid cancer. A meta-analysis of the data was not performed due to the heterogeneity of the methods and risk factor definitions across studies. Notable differences in study characteristics and study samples are indicated on the plot. (TIF) [file pone.0029177.s004.tif]
