# Supplementary material for: BMI, Diet and Female Reproductive Factors as Risks for Thyroid Cancer: A Systematic Review
Source: PLoS One. 2012 Jan 19;7(1):e29177. doi: 10.1371/journal.pone.0029177 (PMC3261873; doi:10.1371/journal.pone.0029177)
Supplement: Table S1 — Summary of reviewed studies on BMI (body mass index) and thyroid cancer. (DOCX) [file pone.0029177.s005.docx]

**Table S1.** Summary of reviewed studies on BMI (body mass index) and thyroid cancer.

| **Reference** | **Study design, sample size, years** | **Exposure and measurement** | **Outcome and measurement** | **Results** | **Strengths and limitations** |
| --- | --- | --- | --- | --- | --- |
| Renehan *et al.* 2008 | Meta-analysis of 2375 cases in women and 1212 in men. Total sample size of 3,303,073 of studies published 1966-2007. Five prospective observational studies from Europe, Australia and Asia-Pacific. | BMI determined at baseline from self-report and direct measurements. | Incident cancer cases during follow-up. | Men: relative risk for every 5kg/m2 increase in BMI for thyroid cancer 1.33 (1.04-1.70). Women: relative risk 1.14(1.06-1.23) | **Strengths**  Checked for publication bias with the use of funnel plots. Prospective studies used so a temporal relationship was established. Assessed the methodological quality of each study included in the analysis. Assessed heterogeneity between studies. |
|  |  |  |  |  | **Limitations**  Some studies used self-reported measures of height and weight which could lead to misclassification. |
| Dal Maso, *et al.* 2000 | Pooled analysis of 12 case-control studies from the United States, Japan, China and Europe.  Includes 2056 female and 417 male cases. 3358 female and 965 male controls. Studies published between 1980 and 1997. | Reported height, weight and BMI at the time of diagnosis and BMI during the late teens (age 17-20). | All types of thyroid cancer but most cases (78%) had papillary carcinoma. | BMI at diagnosis had weak association with thyroid cancer in females (OR 1.2, 95%CI 1.0-14) but not males (OR 1.0, 95%CI 0.8-1.4) when the highest tertile was compared to the lowest. BMI during the late teens was not associated with thyroid cancer in either females (OR 1.1, 95%CI 0.9-1.3) or males (OR 1.4, 95%CI 0.9-2.1). | **Strengths**  Large sample size. Adjusted for age and history of radiotherapy. Tested for heterogeneity by geographic area and age. |
|  |  |  |  |  | **Limitations**  BMI at the time of diagnosis does not provide a temporal relationship between it and thyroid cancer. Did not control for other confounding factors such as physical activity and diet. Possibility for misclassification of exposure information as the information on weight and height was self-reported. |
| Clavel-Chapelon *et al.* 2009 | Prospective cohort, includes 91,909 participants, 317 cases of papillary and follicular thyroid cancer from 1990-2005. Women between the ages of 40 and 65 in 1990 in France, mainly consisted of teachers. | BMI calculated from height and weight as reported through multiple questionnaires. | Papillary and follicular cancers self-reported and then verified by physicians and pathology reports. | For every 5kg/m2 unit increase in BMI the HR for thyroid cancer was 1.20 (95%CI 1.04-1.38). For BMI >24.03 the HR for thyroid cancer was 1.47 (95%CI 1.04-2.08) in comparison to BMI less than 20. For BMI > 30 the HR for thyroid cancer was 1.76 (1.12-2.76) when compared to BMI of 18.5-22. | **Strengths**  Prospective study. Large sample size. Controlled for confounding from multiple risk factors. |
|  |  |  |  |  | **Limitations**  Self-reported measures of BMI may lead to misclassification. Did not control for confounding from radiation. |
| Leitzmann *et al.* 2010 | Prospective cohort of  484,326 participants including 352 incident cases from 1995-2003. Both females and males in the United States who are members of American Association of Retired People (AARP; age 50-71) | Weight and height self-reported in the baseline questionnaire and was used to calculate BMI. | Thyroid cancer cases identified through linkage to the state cancer registries. | People with BMI of 25-29.9 at an increased risk for thyroid cancer in comparison to those with BMI of 18.5-24.9 (RR1.27, 95%CI 0.99-1.64). For those with BMI > 30 the relative risk increased to 1.47 (1.03-2.10). For papillary carcinoma these relative risks were 1.59 (1.18-2.18) and 1.47 (1.03-2.10) respectively. When split by gender in those with BMI >30 an association seen in males (RR 1.89, 95%CI 1.21-2.96) but not in females (RR 1.10, 95%CI 0.75-1.61). | **Strengths**  Large sample size. Prospective study. High follow-up rates. Controlled for confounding from many risk factors. |
|  |  |  |  |  | **Limitations**  Members of AARP may not be generalizable to the rest of the population because of their older age. Weight was measured at baseline and thus might change over the study period possibly leading to misclassification. Self-reported measures of weight and height might also lead to misclassification. |
| Meinhold *et al.* 2010 | Prospective cohort of  90713 participants, 282 incident thyroid cancer cases from 1983-2006 among United States Radiologic Technologists. | Many variables collected through questionnaires including height and weight. | Self-reported or registry obtained thyroid cancer cases. | Women: A BMI >35kg/m2 associated with increased risk of thyroid cancer when compared with normal BMI (HR 1.74, 95%CI 1.03-2.94). Men: association not significant (HR 2.14, 95%CI 0.6-7.67). | **Strengths**  Adjusted for confounding on many risk factors. Good follow-up. |
|  |  |  |  |  | **Limitations**  Self report of risk factors may lead to misclassification of exposure information. May not be generlizable because radiation technologists may be monitored closely for thyroid cancer. |
| Guignard *et al.* 2007 | Case-control study of 332 cases and 412 matched controls from 1993-1999. Residents of New Caledonia living there for at least five years at the time of diagnosis for cases or at the reference year for controls. | Face-to-face interview with a structured questionnaire was used to measure multiple risk factors. BMI was calculated using height and weight at the reference year. | Papillary or follicular thyroid cancer cases identified from pathology laboratories and the New Caledonia cancer Registry. | Women: BMI of 30-34.99 (OR 1.92, 95%CI 1.14-3.22) and >35 (1.85, 95%CI 1.02-3.35) showed increased risk of thyroid cancer when compared to those with BMI between 18.5 and 24.99. By age, women <50 with BMI of 30-34.99 (OR 1.95, 95%CI 0.74-5.17) or >35 (OR 0.92, 95%CI 0.33-2.58) did not see increased risk for thyroid cancer. Women 50 and over did show increased risk with a BMI of 30-34.99 (OR 4.56, 95%CI 1.43-14.54) and >35 (OR 5.51, 95%CI 1.49-20.30). Men: No association between thyroid cancer and BMI >30 (OR 1.04, 95%CI 0.28-3.79). | **Strengths**  Cases selected from pathology labs and cancer registries while controls were selected from electoral rolls so selection bias is not likely. Adjusted ORs for age, year of reference, ethnicity and reproductive factors. |
|  |  |  |  |  | **Limitations**  Height and weight determined through questionnaire with possible recall or misclassification bias. Small sample size in men did not allow for any subgroup analysis within this population. Did not control for many risk factors of thyroid cancer such as radiation exposure. |
| Suzuki *et al.* 2008 | Case-control study of 173 cases and 865 controls from 2001-2005. Patients at the Aichi Cancer Centre Hospital in Nagoya, Japan aged 20-79. | Current weight, weight at age 20 and height were collected through a questionnaire to allow for calculation of BMI. | Thyroid cancer cases who visit the hospital within 6 months of a thyroid cancer diagnosis. | Increased risk for thyroid cancer when the top tertile of current BMI compared to the bottom (OR 1.71, 95%CI 1.06-2.78). When broken down by sex the ORs were 4.21 (1.13-15.70) for males and 1.48 (0.86-2.57) for females. BMI at age 20 associated with a non-significant increased risk of thyroid cancer when comparing top tertile with bottom (OR 1.54, 95%CI 0.97-2.44). | **Strengths**  In a validation analysis, a high correlation was found between self-reported measures of weight and height versus those obtained from charts. Adjusted for many potential confounding factors. Selection bias is not an issue because cases and controls come from the same hospital. |
|  |  |  |  |  | **Limitations**  Did not adjust for radiation exposures. Sample size was small for males thus reducing the power. |
| Brindel *et al.* 2009 | Case-control study of 219 cases, 359 controls from 1984-2003. People born and living in French Polynesia. | Height at interview; weight at age 18, 30, 40, 50 and at time of interview; measures through face-to-face structured questionnaire. BMI calculated from these responses. | Thyroid cancer cases from cancer registry. | Women: increased risk of thyroid cancer in the highest quartile of BMI when compared to the lowest (OR 2.3, 95%CI 1.1-4.7) and with high BMI at age 18 (OR 2.3, 1.2-4.4), 30 (OR 3.1, 95%CI 1.5-6.7) and 40 (OR 3.7, 1.0-13.4).  Men: increased risk for thyroid cancer among those with BMI above the median (OR 7.4, 95%CI 1.3-42.3) for those at age 18 (OR 4.5, 95%CI 1.0-19.2), age 30 (OR 15.6, 95%CI 1.6-153) and age 40 (OR 6.8, 95%CI 0.8-60.5). | **Strengths**  Both cases and controls selected from population based registries so selection bias is not likely a problem. Controlled for confounding on many risk factors. |
|  |  |  |  |  | **Limitations**  Recall bias is possible as exposure information was measured through retrospective questionnaire. There is the possibility of misclassification of exposure status as information was collected based on self-report and people may underreport weight. Authors performed multiple statistical comparisons so they have increased the chance of finding a significant result. The sample size for males was small reducing power. |
