# Supplementary material for: BMI, Diet and Female Reproductive Factors as Risks for Thyroid Cancer: A Systematic Review
Source: PLoS One. 2012 Jan 19;7(1):e29177. doi: 10.1371/journal.pone.0029177 (PMC3261873; doi:10.1371/journal.pone.0029177)
Supplement: Table S2 — Summary of reviewed studies on diet and thyroid cancer. (DOCX) [file pone.0029177.s006.docx]

**Table S2**. Summary of reviewed studies on diet and thyroid cancer.

| **Reference** | **Study design, sample size, years** | **Exposure and measurement** | **Outcome and measurement** | **Results** | **Strengths and limitations** |
| --- | --- | --- | --- | --- | --- |
| Horn-Ross *et al.* 2001 | Case-control study of 608 cases and 558 controls from 1995-1998. Women between 20-74 years of age residing in the San Francisco Bay Area. | Dietary iodine including supplement use collected through in-person structured interviews. Dietary iodine intake calculated based on the information provided. Neutron activation analysis of toenail clippings to measure iodine exposure over a 2-4 week period approximately 1 year before the clipping. | Thyroid cancer cases identified through a Cancer Registry. | *Iodine*  Dietary iodine consumption not related to follicular cancer. Significantly lower nail iodine levels in cases with follicular cancer than controls (P=0.02). Iodine consumption inversely related to papillary cancer risk when highest intake quintile compared to lowest (OR 0.49, 95%CI 0.29-0.84). This association was mostly attributable to multivitamin pills. No significant association between papillary carcinoma and iodine.  *Diet*  Papillary thyroid cancer not related to fish consumption for shellfish (OR 0.93, 95%CI 0.64-1.3), saltwater fish (OR 0.96, 95%CI 0.69-1.3), fresh-water fish (OR 1.1, 95%CI 0.74-1.7) or canned tuna (OR 1.2, 95%CI 0.90-1.7). Cooked seaweed associated with decreased thyroid cancer risk (OR 0.61, 95%CI 0.44-0.84). | **Strengths**  Controlled for confounding of many risk factors. Large sample size. |
|  |  |  |  |  | **Limitations**  Possibility of selection bias as cases were obtained from a cancer registry but controls were obtained through random-digit dialing. Possibility for recall bias as the cases may recall information for questionnaires differently than the controls. Possibility for misclassification of iodine exposure information as the measure for iodine intake was not direct. Usefulness of the nail clipping biomarker is not well known. |
| Kolonel *et al.* 1990 | Case-control study of 191 cases and 442 controls matched on age and sex from 1980-1987. Both female and male residents of Oahu, Hawaii aged 18 years or older. | Multiple risk factors measured through interview. Iodine intake was measured through retrospective recall of diet. | Thyroid cancer cases identified through the Hawaii Tumor Registry (mainly papillary cancer cases). | Females: non-significant OR for thyroid cancer risk in highest iodine intake quartile compared to lowest (OR 1.6, 95%CI 0.8-3.2). Males: OR was 1.3 (0.4-3.7). | **Strengths**  Selection bias not likely as cases selected from a population based registry and controls selected through random sample of the population. |
|  |  |  |  |  | **Limitations**  Recall bias is possible as cases may recall diet information differently than controls. The iodine intake was estimated based on diet and authors did not measure salt intake thus there is a possibility of misclassification of exposure information. Odds ratios were only adjusted for age and ethnicity but not any other confounding factors. |
| Pettersson *et al.* 1996 | Ecological study of 5,838 cases from 1958-1981 using Swedish Cancer Registry. | Iodine deficient and iodine sufficient areas defined on the basis of goiter prevalence. Salt ionization was introduced during this time taking off in 1966 when there was no difference in the prevalence of goiter between these two areas. | Thyroid cancer incidence based on data from the Swedish Cancer Registry. | Age standardized incidence rates increased significantly over time for all types of thyroid cancer and in both regions. Incidence of papillary cancer higher in iodine-sufficient areas than iodine-deficient areas. In both areas age standardized incidence rate of papillary cancer increased 2 fold. After adjusting for age, sex and birth cohort risk of developing papillary carcinoma in iodine deficient area was significantly reduced (RR 0.80, 95% 95%CI 0.73-0.88). Both males and females had higher risk of developing follicular cancer in iodine deficient region (M: RR 1.98, 95%CI 1.6-2.4; F: RR 1.17, 95%CI 1.04-1.32). | **Strengths**  Cancer registry is almost complete reducing the possibility of selection bias. |
|  |  |  |  |  | **Limitations**  There is a possibility of misclassification of exposures status as iodine-deficient areas were defined by areas that previously had high rates of goiter. Did not control for many relevant confounding factors. Ecological study so population level associations cannot be inferred to the individual. |
| Williams *et al.* 1977 | Ecological study of 108 cases in Iceland and 103 cases in Northeast Scotland from 1944-1963. | One region with high dietary iodine intake in Iceland and one region of average dietary iodine intake in northeast Scotland. | Incidence and histological type of thyroid cancer in both regions. | The age-specific incidence of papillary carcinoma was five times higher in Iceland than Northeast Scotland across all groups. In Iceland papillary carcinoma accounted for 71% of all thyroid cancers but only 4% in northeast Scotland. In people over 65 the incidence of undifferentiated carcinomas in Iceland was twice that of North East Scotland. | **Strengths**  Both populations have broadly similar ethnic background and standards of medical care. All thyroid carcinomas were examined by the same two pathologists. |
|  |  |  |  |  | **Limitations**  No statistical tests were performed. Did not control for confounding. Difficult to make comparisons between two populations as the populations can differ on many different factors not just iodine intake. Ecological study so population level associations cannot be inferred to the individual. |
| Harach *et al.* 2008 | Ecological study of residents of the province of Salta, Argentina from 1958-2007. | Pre-prophylaxis period includes cases diagnosed 5 years before and 10 years after salt iodination (1958-1972). Post-prophylaxis period (1985-2007) | Incidence of thyroid cancer. | Significant increase in papillary carcinoma (P<0.001) and decrease in undifferentiated carcinoma (P<0.2) between two time periods. The ratio of papillary to follicular carcinoma increased from 1.7:1 to 3.9:1. The overall incidence rates for thyroid cancer showed a progressive increase (1.6/ 100,000 in 1960 to 3.6/100,000 in 2001) especially for papillary cancer. | **Strengths**  Pre and post iodination periods examined. |
|  |  |  |  |  | **Limitations**  Did not control for confounding factors that may have changed over time such as standards of medical care, environmental exposures, and pathological criteria. Did not take into account 1988 WHO changes in histopathology classification favoring the diagnosis of the papillary subtype at the cost to follicular carcinoma. Did not describe acquisition of incidence in detail and therefore it is not known if there is selection bias. Did not provide sample size of population. Ecological study so population level associations cannot be inferred to the individual. |
| Bosetti *et al.* 2001 | Pooled analysis of 13 case-control studies of 2497 cases and 4337 controls from studies published between 1980 and 1997, from US, Japan, China and Europe. | Food frequency questionnaires for total fish, salt water fish and shellfish consumption. | Thyroid cancer. | No significant association with thyroid cancer found for high total fish consumption in comparison to low consumption (OR 0.88 95%CI 0.71-1.1). In the endemic goiter region OR was 0.65 (0.48-0.88) and in iodine rich areas was 1.1 (0.85-1.5). For salt water fish consumption the OR was 1.1 (0.82-1.4) and for shellfish consumption it was 1.0 (0.80-1.3). Pattern of risk consistent when data was restricted to women and papillary cancer. | **Strengths**  Large sample size. Adjusted for confounding from history of thyroid disease and history of radiation. Evaluated heterogeneity of the results based on relation to endemic goiter areas and quality of the questionnaires. |
|  |  |  |  |  | **Limitations**  Possibility for recall bias as exposure information gathered through questionnaire. Exposure information collection across the original studies was not homogeneous. |
| Memon *et al.* 2002 | Case-control study of 313 matched cases and controls from 1981-1999 and includes residents of Kuwait during the data collection period aged < 70 years old. | Structured questionnaire asking information on many possible risk factors including 13 dietary items. | Thyroid cancer from cancer registry information and charts from the Kuwait Cancer Control Centre. | Protective effect found for high consumption of fresh fish (OR 0.5, 95%CI 0.4-0.7). Processed, canned or frozen fish increased risk of thyroid cancer (OR 2.2, 95%CI 1.6-3.0). No association found with total fish consumption (OR 0.6, 95%CI 0.3-1.0) or shellfish consumption (OR 1.2, 95%CI 0.7-2.0). Increased risk found with high consumption of chicken (Or 1.7, 95%CI 1.2-2.3). No association found with the consumption of green vegetables (OR 0.8, 95%CI 0.6-1.4). Increased risk of thyroid cancer with the consumption of cabbage (OR 1.9, 95%CI 1.1-3.3) and cauliflower (OR 1.8, 95%CI 1.0-3.2) but not with the consumption of Brussels sprouts (OR 0.7, 95%CI 0.1-4.3) or Broccoli (OR 0.9, 95%CI 0.2-3.6). | **Strengths**  Controlled for confounding variables. |
|  |  |  |  |  | **Limitations**  Did not state what confounding variables were controlled for. Possibility for selection bias as cases selected from a thyroid cancer clinic and controls selected from local primary healthcare clinics. Possibility for recall bias as exposure information was obtained through a retrospective questionnaire. Performed multiple statistical tests increasing the probability of getting a significant result by chance. |
| Mack *et al.*2002 | Case-control study of  292 cases and 292 matched controls from 1980-1983. Los Angeles County women aged 15-54, English speaking and white. | In person or telephone interview using a structured questionnaire for multiple thyroid cancer risk factors. | Thyroid cancer cases as identified through the Los Angeles County population based registry. | Risk of thyroid cancer not related to shellfish consumption (OR 0.8, 95%CI 0.3-2.4), saltwater fish consumption (OR 0.6, 95%CI 0.3-1.3) or freshwater fish consumption (OR 0.6, 95%CI 0.2-1.7) in high intake group compared to low intake group. Decreased risk of thyroid cancer with consumption of turnips or rutabagas a few times a year for all types of thyroid cancer (OR 0.6, 95%CI 0.3-1.0) and papillary thyroid cancer (0.5, 95%CI 0.2-0.9). Increased risk for papillary thyroid cancer was in those who had more than 10 years use of multivitamin at least weekly (OR 2.9, 95%CI 1.2-7.4). | **Strengths**  Eighty percent of eligible cases participated in the study. Matched controls on birth year, race and neighborhood. Adjusted for benign thyroid conditions and radiation as possible confounders. |
|  |  |  |  |  | **Limitations**  Selection bias possible as controls selected from neighborhood and cases selected from registry. Possible recall bias as exposure information was collected through retrospective interviews. Interviewers not blinded leading to possible interviewer bias. Sample sizes varied depending on question leading to lower power for some tests. Multiple statistical comparisons performed increasing the chance of getting statistically significant responses. |
| Bosetti *et al.* 2002 | Pooled analysis of 11 case-control studies from US, Asia and Europe of 2241 cases and 3716 controls, published between 1980 and 1997. | Food frequency questionnaire for total cruciferous and other vegetable intake. | Thyroid cancer. | No significant association found for thyroid cancer and high total cruciferous vegetable consumption compared to low (OR 0.94, 95%CI 0.80-1.10). Results were similar when researchers looked at iodine rich areas, endemic goiter areas, women and papillary carcinoma. For vegetables other than cruciferous inverse relationship found with thyroid cancer in high consumption versus low (OR 0.82, 95%CI 0.69-0.98) | **Strengths**  Large sample size. Adjusted for confounding from history of thyroid disease and history of radiation. Evaluated heterogeneity of the results based on relation to endemic goiter areas and quality of the questionnaires. |
|  |  |  |  |  | **Limitations**  Possibility for recall bias as exposure information gathered through questionnaire. Exposure information collection across the original studies was not homogeneous. |
| Markaki *et al.* 2003 | Case-control study of  113 cases and 138 controls matched on age, gender and hospital from 1990-1993 of male and female patients from Greece. | Patients asked through a food frequency questionnaire about their average lifetime consumption of each food item prior to a diagnosis of thyroid cancer. | Thyroid cancer cases from 2 of the major hospitals in Athens, Greece. | Increased consumption of pork increased risk of thyroid cancer (OR 1.63, 95%CI 1.13-2.35). Decreased risk for thyroid cancer with increased raw tomato (OR 0.92, 95%CI 0.85-0.99) and lemon (OR 0.91, 95%CI 0.85-0.98) consumption. Significant dose response relationship found for fresh tomatoes (P =0.002) and pork (P=0.001). Decreased risk for all thyroid cancer found with the consumption of fruits (OR 0.68, P=0.01), raw vegetables (OR 0.71, P=0.02) and mixed vegetables and fruits (OR 0.73, P=0.04). Increased risk for follicular thyroid cancer found with increased consumption of fish and cooked vegetables (OR 2.79, P=0.02). | **Strengths**  Cases and controls both selected from the same hospitals so selection bas should not be an issue. Interviewers blinded to case status. Controlled for confounding by matching on age, gender and health unit (equivalent to SES). Performed factor analysis which addresses the issues of multiple comparisons. 100% response rate. |
|  |  |  |  |  | **Limitations**  Recall bias still a possibility because exposure status measured through a retrospective questionnaire. Did not report confidence intervals for odds ratios of factor analyses. |
| Fioretti *et al.* 1999 | Case-control study of  339 cases and 617 controls from 1986-1992 of patients between the ages of 15 and 75 who resided in Northern Italy. | Interviews conducted using structured questionnaires on multiple risk factors. Diet measured through a food frequency questionnaire. | Thyroid cancer cases who were admitted to one of eight major hospitals. | Increased thyroid cancer risk was associated with high refined cereal intake (OR 2.0, 95%CI 1.4-2.9) and low beta-carotene intake (OR 1.4, 95%CI 1.0-1.9). | **Strengths**  Large sample size. Selection bias not likely as controls were collected from the same general population as the cases. Controlled for confounding from multiple risk factors. |
|  |  |  |  |  | **Limitations**  Possibility for recall bias as dietary information gathered through interviews. |
| Horn-Ross *et al.* 2002 | Case-control study of 608 cases and 558 controls from 1995-1998, includes women between the ages of 20 and 74 who were residents of the San Francisco Bay Area. | Multiple risk factors measured through in-person interviews using standardized structured questionnaires. Dietary intake measured through a food frequency questionnaire to determine phytoestrogen intake. | Thyroid cancer cases identified through the Greater Bay Area Cancer Registry. | Reduced risk of thyroid cancer found with high tofu consumption (OR 0.50, 95%CI 0.30-0.84), moderate consumption of soy-burgers and meat substitutes (OR 0.51, 95%CI 0.29-0.89) and high consumption of alfalfa sprouts (OR 0.62, 95%CI 0.41-0.93). An increased consumption of total phytoestrogens associated with decreased risk of thyroid cancer (OR 0.62, 95%CI 0.39-0.99). | **Strengths**  Controlled for many possible confounding factors including radiation, family history, and reproductive factors. Large sample size. |
|  |  |  |  |  | **Limitations**  Cases selected through a cancer registry and controls selected through random digit dialing leading to the possibility of selection bias. Possibility for misclassification of exposure status as not all dietary phytoestrogen sources measured. |
