# Supplementary material for: BMI, Diet and Female Reproductive Factors as Risks for Thyroid Cancer: A Systematic Review
Source: PLoS One. 2012 Jan 19;7(1):e29177. doi: 10.1371/journal.pone.0029177 (PMC3261873; doi:10.1371/journal.pone.0029177)
Supplement: Table S3 — Summary of reviewed reproductive factor studies and thyroid cancer. (DOCX) [file pone.0029177.s007.docx]

**Table S3.** Summary of reviewed reproductive factor studies and thyroid cancer.

| **Reference** | **Study design, sample size, years** | **Exposure and measurement** | **Outcome and measurement** | **Results** | **Strengths and limitations** |
| --- | --- | --- | --- | --- | --- |
| La Vecchia *et al.* 1999 | Pooled analysis of 13 case-control studies published between 1980 and 1997 from USA (4 studies), Asia (2 studies), and Europe (7 studies), and examining a total of 2132 female cases and 3301 female controls. | Variables included ever use, total duration of use, age at first and last use for OC and HRT, and ever use of fertility drugs, hormones for menstrual irregularities, and lactation suppression. | Data source defined by individual studies. | *Oral contraceptives (OC)*  Ever use of OCs was considered among 13 studies but showed a non-significant elevated risk of thyroid cancer (OR 1.2, 95%CI 1.0-1.4). Eight studies had ORs above 1. There was no association with duration of use, age at first use, or use before first birth, nor was there a difference across geographic regions. There was a significantly decreasing risk with increasing age for ever use and duration of use, as well as rising risk with increasing age for age at first use.  Risk for current users of OCs was 1.5 (95%CI 1.0-2.1) and declined to 1.1 after 10 years of stopping OC use.  *HRT*  No association with HRT use (OR 0.8, 95%CI 0.6-1.1) or duration of HRT use, although risk was lower for women who started HRT after age 50.  No differences across geographic areas, but ORs for ever use of HRT, duration and age at first use decreased with increasing age at diagnosis.  *Fertility drugs*  No association with fertility drugs (OR 1.6. 95%CI 0.9-2.9).  *Lactation suppression*  Lactation suppression treatment was associated with higher risk of thyroid cancer (OR 1.5, 95% CI 1.1-2.1) and was higher at younger age at diagnosis. | **Strengths**  Analysis adjusted for study, age, history of radiation and parity (and for HRT use, menopause status and the type of menopause (i.e. natural or artificial)). Large proportion (40%) of subjects used OCs, adding greater power to the analysis on OC use and thyroid cancer risk. Risk with OC use was stronger for papillary cancers (OR=1.6) than for other histologic types. Authors suggest that increased surveillance among OC users may lead to higher detection rates. |
| Negri *et al.* 1999 | Pooled analysis of 14 case-control studies published between 1980 and 1997, from USA (4 studies), Asia (2studies), and Europe (8 studies), and examining a total of 2247 female cases and 3699 female controls. | Variables included age at menarche, menopausal status, age at menopause, natural or artificial menopause, history and age at hysterectomy monolateral or bilateral oophorectomy, number of pregnancies, births, miscarriages and induced abortions, outcome of first pregnancy, age at first and last pregnancy and birth, number of breast fed children, duration of breast feeding and history of infertility. | Data source defined by individual studies. | *Menopause*  Among menstrual factors, artificial postmenopausal status was significantly associated with thyroid cancer risk (OR 1.8, 95%CI 1.4-2.4). Stratification by carcinoma type showed that follicular was more strongly associated with artificially-induced menopause (OR 3.1, 95%CI 1.7-5.6). Natural menopause was not significantly associated with thyroid cancer (OR 1.3, 95%CI 1.0-1.8).  *Menarche*  There was a small and non-significant association between late menarche and thyroid cancer risk (OR 1.04, 95%CI 1.0-1.1)  *Births*  Only miscarriage at first pregnancy was significantly associated with risk (OR 1.8, 95%CI 1.2-2.6). Stratification by carcinoma type showed that papillary was more strongly associated with miscarriage (OR 1.7, 95%CI 1.1-2.7). | **Strengths**  Analysis adjusted for study, age, history of radiation exposure, use of HRT, OC use, and parity.  **Limitations**  Despite pooling data, results show only weak associations for most factors. The association with artificial menopause may be due to greater surveillance of women who undergo surgical menopause to monitor for hormonal changes. |
| Navarro Silvera *et al.* 2005 | Prospective cohort of women aged 40-59 recruited into the Canadian National Breast Screening Study. 89,835 women enrolled, 1980-2000. | Menstrual and reproductive history was assessed by a questionnaire administered at enrollment. | Incident cases of thyroid cancer and deaths from all causes were ascertained from the Canadian Cancer Database and the National Mortality Database. | There was no evidence of altered thyroid cancer risk from any menstrual, reproductive or hormonal factor. This included age at menarche, ever being pregnant, number of pregnancies or births, age at first birth, menopausal status and use of oral contraceptives or HRT. | **Strengths**  Population-based cohort. No recall bias because reproductive history was collected at baseline. Long follow-up of cohort (16 years on average).  **Limitations**  Small number of thyroid cancer cases identified (169 cases). Menopausal status only collected at baseline, not at time of diagnosis. |
| Neale *et al.* 2005 | Record-linkage cohort study of women who had delivered a baby in Sweden between 1961 and 1996. 1,234,967 women. | Birth information, including parity of the mother and whether the births were of twins, were obtained from the Civil Birth Register of Sweden. | 986 cases of thyroid cancer were identified from the Swedish Cancer Registry. | No significant change in risk was seen with increasing number of pregnancies, whether the children were twins nor with age at first birth. | **Strengths**  Population based cohort. No recall bias because childbirth history was collected from national birth register.  **Limitations**  Couldn’t control for confounding from miscarriages, hormone use or lifestyle factors. Analysis only included women who had been parous. |
| Hannibal *et al.* 2008 | Case-cohort study of 54,362 women with fertility problems from Danish clinics; 29 cases of thyroid cancer; 1963-1998. | Types of fertility drugs and number of cycles of use, surgical interventions, cause of infertility, BMI, OC use, age at menarche | Hospital records | Clomiphene use: ever (OR 2.29, 95%CI 1.08-4.82); use in parous women (OR 3.76, 95%CI 1.48-9.58).  Ever use of progesterone use (OR 10.14, 95%CI 1.93-53.34)  No association with gonadotropins | **Strengths**  Adjusted for age at first birth  **Limitations**  Small number of cases. |
| Rosenblatt *et al.* 2009 | Prospective cohort of 20 cases of thyroid cancer among Shanghai female textile workers, 1998-1991. | Interview with standardized questionnaire on menstrual and reproductive history, and contraceptive use (current and previous use, duration of use, type of OC). | Shanghai Cancer registry and linked to vital statistics registry. | Ever use of OC (OR 0.75, 95% CI 0.46-1.23).  No association with duration of OC use and risk of thyroid cancer. | **Strengths**  Adjusted for parity and age.  **Limitations**  Study was not specific to thyroid cancer (examined 9 cancers types) and therefore did not control for all confounders for thyroid cancer. |
| Dorjgochoo *et al.* 2009 | Prospective cohort of women aged 40-70 years followed for a median of 7.5 years; 83 cases of thyroid cancer in 2250 women during 2000-2007. | Interviews at 3 occasions during follow-up on contraceptive use (oral, injection, IUD, tubal sterilization), including duration of use, age at first use and time since last use. Also asked about menstrual and reproductive history to control for in analysis. | Shanghai Cancer registry and linked to vital statistics registry. | Ever use of OC (OR 0.63, 95%CI 0.38-1.04).  IUD use negatively associated with thyroid cancer risk, but this was not significant.  Tubal sterilization showed no association. | **Strengths**  Adjustment for age at menarche, number of live births, menopausal status, family history of cancer.  **Limitations**  Small number of women and relatively short follow-up time. |
| Pham *et al.* 2009 | Cohort study of women aged 40-79 living in 24 areas of Japan with cancer registries, includes 37,986 women from 1988-1997. | Information on menstrual items, reproductive items and hormone use was obtained using a baseline self-administered questionnaire. | 86 new cases of thyroid cancer were identified from the local cancer registry. | No significant association for any reproductive factor. Age at menarche (p=0.33); ever pregnant (HR 0.56, 95%CI 0.25-1.24) vs. never; number of pregnancies (p=0.96); age at first birth (p=0.68); age at menopause (p=0.98); and use of hormone drugs (HR 0.67, 95%CI 0.21-2.12) vs. never use. | **Strengths**  Large cohort study. Information collected at baseline. Cases selected from cancer registries.  **Limitations**  A cancer registry was available in only 24 of 45 areas of Japan. Menstrual and reproductive factors were assessed through a self-reported questionnaire. A small number of cases of thyroid cancer were diagnosed. |
| Rossing *et al.* 1998 | Case-control study of 410 women with papillary cancer and 574 population-based controls, age 18-64 in Washington State, 1988-94. | Telephone interview on demographics, lifestyle characteristics, medical history, family history of cancer and thyroid disease, reproductive history (menstrual, pregnancy, contraceptive and non-contraceptive hormones use). | Cancer Surveillance System population registry. | Among women 45-64, no association of papillary with OCs or HRT.  Among women under 45, reduced risk with ever use of oral contraceptives(OR 0.6, 95%CI 0.4-0.9) but no other associations with OCs or HRT. | **Limitations**  Information on OC use was based on recall. More women in the control declined to participate suggesting some participation bias. |
| Mack *et al.* 1999 | Case-control study of women aged 15-54 years at the time of diagnosis, living in Los Angeles County. 292 paired cases and controls, 1980-1983. | Menstrual and reproductive history was collected through telephone interviews. | Thyroid cancer cases were identified through the University of Southern California Cancer Surveillance Program. Age, sex and neighborhood matching was done by conducting neighborhood searches. | An increased risk was observed for women who had a hysterectomy (OR 1.9, 95%CI 1.0-3.8) or complete oophorectomy (OR 6.5, 95%CI 1.1-38.1), compared to women still menstruating.  An increased risk was seen among women who used lactation suppressants and the number of live births (p=0.03).  No significant risk was observed for age at menarche, irregular menstrual cycles, ever being pregnant, number of live births, age at first birth, natural menopause or oral contraceptive use. | **Strengths**  Controls were selected through neighborhood matching.  **Limitations**  Information on reproductive history was collected through an interview and could introduce recall bias. |
| Rossing *et al.* 2000 | Case-control study of 410 women with papillary thyroid cancer and 574 hospital controls age 18-64 in Washington State, 1988-1994. | Questionnaire on menstrual and reproductive history, medical history, use of exogenous hormones, family history of cancer, body size, diet, alcohol consumption, smoking, demographics. | Cancer Surveillance System population registry. | Among women 45-64, no association of papillary with number of live births, age at first live birth, age at last live birth, or breastfeeding.  Among women under 45, no association with number of live births, age at first or last birth, lifetime duration of lactation.  Time since last birth (1-5yrs): OR 1.8, 95%CI 1.0-3.2.  Number of births (past 5 yrs): OR 1.6 (95%CI 1.0-2.7) for 1 birth; OR 4.2 (95%CI 2.0-8.9) for >1.  Time since lactation (<1yr):OR 2.5 (95%CI 1.1-5.5).  Duration of lactation (past 5 yrs): OR 2.9 (95%CI 1.5-5.5) for >12 months.  Increasing or above average weight linked non-significantly to higher risk, pointing to possible connection with hormone balance. | **Limitations**  Information on reproductive history was based on recall. Women who had been pregnant may have had more contact with physicians, leading to more detection. |
| Memon *et al.* 2002 | Case-control study of men and women diagnosed with thyroid cancer in Kuwait, including 238 cases among women and matched controls, 1981-1996. | Gynecologic and reproductive history collected through structured interview. | 238 cases of thyroid cancer identified in the Kuwaiti Cancer Registry of the Kuwait Cancer Control Centre. Controls were selected from among people visiting a primary care clinic for minor care or other reasons. | A significant increase in thyroid cancer risk was seen with increased age at last birth (p<0.05), but not with age at first birth. There was a significant decrease in risk with an increasing number of miscarriages (p<0.05). There were no significant associations with age at menarche, ever being pregnant, number of pregnancies, menopausal status, age of menopause, and use of any hormones. | **Strengths**  Authors initially identified all thyroid cancer cases in the country. Age, gender and geographic matching.  **Limitations**  Information on reproductive history was based on recall.  More women in the control group declined to participate, suggesting some participation bias. |
| Sakoda *et al.* 2002 | Case-control study of women aged 20-74 diagnosed with thyroid cancer in the Greater Bay Area of California. 608 cases and 558 controls. 1992-1998. | Information was collected on a variety of factors through interviews using a standardized structured questionnaire. | Cases were identified through the Greater Bay Area Cancer Registry; controls were identified through random digit dialing and were matched by age and race. | A significant risk was seen when the age of menarche was younger than 12 (OR 1.5, 95%CI 1.1-2.3) compared to women aged 12.  A significant increase in risk was seen in women having been pregnant twice (OR 1.7, 95%CI 1.1-2.7) compared to never being pregnant.  There was a significant increase in risk with an older age of first pregnancy. Oral contraceptive use was associated with a decrease in risk of thyroid cancer (OR 0.73, 95%CI 0.52-0.97).  There was no associated risk with ever being pregnant, menopausal status and use of HRT. | **Limitations**  Information on reproductive history was based on recall.  More women in the control group declined to participate, suggesting some participation bias. |
| Fernandez *et al.* 2003 | Case-control study of women who were users of HRT age 45-79 in Italy between 1983-1999 from several case-control studies, including 65 thyroid cases and 6976 controls of the same age. | HRT usage using questionnaire. | Data source defined by individual studies. | *Hormone replacement therapy*  Ever use: OR 0.8 (95%CI 0.3-2.3)  <2 years of use: OR 1.0 (95%CI 0.3-2.4)  >2 years of use: OR 0.5 (95%CI 0.1-4.0) | **Strengths**  High participation rates among cases and controls.  **Limitations**  Low rates of HRT use in the study population, which limited the statistical power. Study was not specific to thyroid cancer and therefore did not control for all confounders of thyroid cancer. Information on HRT use was based on recall. |
| Zivaljevic *et al.* 2003 | Case-control study of 204 cases and 204 hospital controls age 10+, matched by age, place of residence and time of hospitalization in Yugoslavia, 1996-2000. | Interview with standardized questionnaire on thyroid gland diseases, diagnostic procedures, radiation and chemical exposures, medication use, family history of thyroid disease, reproductive factors (age at menarche, regularity of menstrual cycle, number of pregnancies, outcome of first pregnancy, age at first birth, abortions, parity, age at menopause, use of OCs, estrogen use, thyroid gland enlargement in pregnancy). | Surgery and oncology centers medical records. | Spontaneous abortion: OR 1.89 (95%CI 1.03-3.50)  OC use: OR 2.34 (95%CI 1.31-4.18)  Thyroid enlargement during pregnancy: OR 16.44 (95%CI 3.81-70.80)  When results were adjusted for factors associated with thyroid cancer (e.g. history of residence in high endemic goiter area; radioactive iodine therapy), results were no longer significant. | **Limitations**  Information on reproductive history was based on recall. All controls had a history of rheumatology disease. |
| Truong *et al.* 2005 | Case-control study of 293 women age >18 years with thyroid cancer and 354 age matched population controls from 1993-1999 in New Caledonia. | Questionnaire on sociodemographics, diet, alcohol, smoking, gynecologic and reproductive history, medical conditions, x-ray exposure, occupational and residential history, family history of thyroid cancer. | Pathology records at labs. | Thyroid cancer associated with:  Irregular menstruation: OR1.9 (95%CI 1.2-3.2) for all ages; OR 3.6 (95%CI 1.7-7.4) for age <45 years; OR 3.0 (95%CI 1.3-6.7) for Melanesian ethnicity.  Live births: OR 2.2 (95%CI 1.1-4.3) for >=8. Dose-response trend with number of live births but only significant for more than 8 births.  Voluntary abortion: OR 3.1 (95%CI 1.5-6.2) for ever had an abortion; OR 3.3 (95%CI 1.3-8.4) for age <45 years. Voluntary abortion was more strongly associated with papillary microcarcinomas and follicular carcinomas of all sizes.  No association with: oral contraceptives; HRT; age at menarche; hysterectomy.  Marginally significant: miscarriage (OR 2.3, 95%CI 1.0-5.6) | **Limitations**  Information on reproductive history was based on recall. Study region has high screening rates, suggesting some surveillance bias. |
| Brindel *et al*. 2008 | Case-control study of patients with thyroid cancer diagnosed before the age of 56, born and living in French Polynesia. 201 women matched to 324 controls from 2002-2004. | Information on gynecologic and reproductive history was collected through face-to-face interviews. | Prevalent cases of thyroid cancer were identified from the cancer registry of French Polynesia, medical insurance files and from four endocrinologists in Tahiti. | Significant increased risk observed with having more children (p=0.03).  Significant risk associated with women who underwent artificial menopause compared to women still menstruating (OR 4.5, 95%CI 1.7-12.0).  There was no risk associated with women having experienced natural menopause. There was also no risk for age at menarche, irregular menstrual cycles, ever being pregnant, age at first birth or having had a miscarriage. | **Strengths**  Controls were selected randomly from birth registries and matched by age and sex.  **Limitations**  Participation rates for controls were less than for cases, indicating participation bias. Information on reproductive history was based on recall. |
